# Insight into prostate cancer osteolytic metastasis by RelB coordination of IL‐8 and S100A4

**DOI:** 10.1002/ctm2.70058

**Published:** 2024-10-16

**Authors:** Wenbo Sun, Kenny Xu, Xiao Li, Peipei Qian, Fan Xu, Yanyan Zhang, Xiumei Wang, Zhi Xu, Jiaji Ding, Xinyu Xu, Xiaowei Wei, Qin Jiang, Yong Xu

**Affiliations:** ^1^ Affiliated Eye Hospital Nanjing Medical University Nanjing China; ^2^ Affiliated Cancer Hospital Nanjing Medical University Nanjing China; ^3^ Department of Thoracic Surgery The First Affiliated Hospital Nanjing Medical University Nanjing China; ^4^ College of Medicine Bowling Green Campus University of Kentucky Bowling Green Kentucky USA; ^5^ Department of Oncology Nanjing First Hospital Nanjing Medical University Nanjing China; ^6^ Jiangsu Key Lab of Cancer Biomarkers Prevention, and Treatment Nanjing Medical University Nanjing China; ^7^ Department of Toxicology and Cancer Biology & Markey Cancer Center University of Kentucky Lexington Kentucky USA

**Keywords:** IL‐8, osteolytic metastasis, prostate cancer, RelB, S100A4

## Abstract

**Background:**

Although RANK‐LRANK interaction is essential for osteoclastogenesis, the mechanisms by which cancer cells invade bone tissues and initiate osteolytic metastasis remain unclear. Here, we show that the hyperactivation of RelB fosters prostate cancer (PCa) osteolytic metastasis by coordinating interleukin‐8 (IL‐8) and calcium‐binging protein A4 (S100A4).

**Methods:**

The factors promoting PCa bone metastasis were investigated in sera from PCa patients and tumour tissues derived from nude mice using immunohistochemical analysis and enzyme‐linked immunosorbent assays (ELISA). Cell mobility and mineralization were quantified using BioStation CT and Osteolmage assay. The relative cistrome was investigated in advanced PCa cells by standard transcriptional analyses, including the luciferase reporter response, site‐directed mutagenesis, and chromatin immunoprecipitation (ChIP) assay. PCa cell‐initiated tumour formation, expansion, and bone metastasis were validated in mice using multiple approaches, including orthotopic, intraskeletal, and caudal arterial implantation models.

**Results:**

IL‐8 and S100A4 correlated with patient Gleason scores and bone metastasis. RelB upregulated IL‐8, facilitating androgen receptor (AR)‐independent growth. RelB‐Sp1 interaction enhanced epithelial‐mesenchymal transition (EMT) by activating Snail and Twist. RelB‐NFAT1c super‐enhancer upregulated S100A4 in the organization of the cytoskeleton and bone metastasis. The RelB‐IL‐8‐S100A4 signalling axis was confirmed to promote osteolytic metastasis in nude mice.

**Conclusion:**

RelB‐IL‐8 reciprocally promoted EMT by activating inflammatory signalling and inactivating AR signalling. IL‐8 is essential for provoking PCa metastasis but insufficient to drive bone metastasis. IL‐8‐S100A4 cooperation was necessary for metastatic cells to target the bone.

**Highlights:**

- RelB activates inflammatory signalling by upregulating IL‐8 and suppressing AR.
- RelB upregulates S100A4 by cooperating with NFATC1.
- IL‐8 boosts EMT by activating Snail 1 and Twist 1, and S100A4 exacerbates osteolytic metastasis via calcium consumption.
- RelB harnesses IL‐8 and S100A4 to drive PCa osteolytic metastasis.

## Graphical abstract

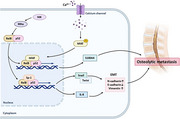

## 1 INTRODUCTION

Bone metastasis is a lethal malignant progression leading to spinal cord compression, skeletal fracture, and bone destruction.^1^, ^2^, ^3^ The integration of invading tumour cells and skeletal microenvironment promotes the development of osteoblastic, osteoclastic, or combined both.^4^, ^5^, ^6^ Activation of receptor activator of NF‐κB (RANK) and its ligand RANKL is essential for developing osteoclastogenesis and osteoclastic metastasis characterized by net bone loss, which is downregulated by osteoprotegerin (OPG).^7^, ^8^, ^9^ Parathyroid hormone‐related proteins derived from tumour cells can stimulate RANK‐mediated cytokine and chemokine activation and contribute to osteoclast differentiation, cell fusion, bone resorption, and cytoskeleton reorganization.^10^, ^11^ However, the precise mechanisms underlying osteolytic metastasis remain poorly understood.

Prostate cancer (PCa) patients frequently develop bone metastasis. The androgen receptor (AR) sustains the growth of both androgen‐dependent and androgen‐refractory (PCa).^12^, ^13^ AR reactivation sustains the progression of androgen‐independent PCa via AR amplification, alternative splicing, or stimulation by other ligands.^14^ However, the AR‐null malignant phenotype is another concern for malignant PCa.^15^ The re‐expression of ARs was shown to inhibit AR‐null cell mobility, indicating that the AR suppresses PCa malignancy by some mechanisms.^16^ In this regard, multiple signalling pathways that sustain PCa progression after AR function declines have been discovered. For instance, NF‐κB and Stat3 participate in promoting malignant castration‐resistant prostate cancer (mCRPC) progression and therapeutic resistance.^17^, ^18^

Nuclear factor (NF)‐κB‐activated proinflammatory signalling is critical for the progression of malignant PCa. Among the stimulated cytokines and chemokines, interleukin (IL)‐8 (*CXCL8*) is critical for exacerbating malignant progression by activating EMT.^19^ Previously, we showed increases in IL‐8 as the prostate‐specific antigen (PSA) decreased in aggressive PCa, suggesting that IL‐8 contributes to PCa growth and metastasis by inactivating ARs.^20^ Furthermore, calcium and calmodulin foster PCa malignant progression by increasing inflammation, particularly in bone tropism.^21^ Notably, calcium‐blinding protein S100A4 released from cancer cells is critical to drive osteolysis.^22^, ^23^

Here, we found that RelB, a noncanonical NF‐κB pathway member,^24^ is necessary for developing PCa cell‐provoked osteolytic lesions. RelB functions as a master regulator, promoting IL‐8‐enhanced AR‐independent cell proliferation, Snail/Twist‐activated EMT processes, and S100A4‐provoked osteolytic metastasis. Notably, IL‐8 (8.3 kDa) and S100A4 (12 kDa), small cell‐secreted proteins, can be easily quantified in patient serum, suggesting that they may serve as useful diagnostic biomarkers for the early detection of PCa bone metastasis beyond PSA screening.

## 2 MATERIALS AND METHODS

### 2.1 Cell culture and gene manipulation

American Type Culture Collection (ATCC) provided all PCa relative cell lines used in this study, including human AR‐positive cell lines (LNCaP, 22Rv1, and C4‐2B), the human AR‐null cell lines (PC‐3 and DU‐145), the murine AR‐null PCa cell line RM‐1, the human normal prostate epithelial cell lines (PrEC and PZ‐HPV‐7), and the human osteoclast precursor cell line RAW264.7. Cells were cultured in the suggested medium and conditions. Relative protein expression was manipulated in PCa cells by ectopically expressing the cDNAs or open reading frames (ORFs) of human *RelB* (Addgene), *IL‐8* (Origene), *S100A4* (OriGene), and *NFATc1* (Addgene) in LNCaP cells using Lipofectamine 2000 reagent (Thermo Fisher). Conversely, RelB, IL‐8, Snail 1, Twist 1, NFATc1, and S100A4 were knocked down in PC‐3 and RM‐1 cells by transducing a lentiviral pGLPZ vector carrying relative shRNA duplexes (Open Biosystem). Neomycin and puromycin were used to select stable cell clones.

### 2.2 Cell treatment

PCa cells were cultured in a completed medium with 10 ng/mL TNF‐α (219‐TA‐005, R&D Systems) or 5 µM parthenolide (PTL) (512732, Sigma) to induce or inhibit RelB function. In addition, PCa cell culture included 50 ng/mL phorbol 12‐myristate 13‐acetate (PMA; P1585, Sigma) or 5 µM tacrolimus (FK506) (F4679, Sigma) to induce or inhibit NFATc1 function.

### 2.3 Cell mineralization

The level of cell mineralization was analyzed using an Osteolmage Assay Kit (PA‐1503, Lonza). In brief, 10^5^ PCa cells were plated in 12‐well plates and cultured for 12 h. After faxing with ethanol, the cells were stained for 30 min. After rinsing with 1× phosphate‐buffered saline (BPS), the cells were imaged using a fluorescence microscope or quantified at 490/520 nm, respectively.

### 2.4 Cell migration and invasion

For the real‐time monitoring of living cell motility, the cell culture dishes were placed in a BioStation CT equipped with an autofocus mechanism that allowed the capture of in‐focus images (Nikon). Multiple points of cell imaging traces were observed over 48 h. The distance of cell movement was automatically calculated regardless of the direction of the cell movement. Cell invasion was analyzed using a Transwell assay kit (CBA‐100‐C, Cell Biolabs). Briefly, 10^4^ cells were plated into the upper chambers with 300 µL serum‐free medium settled in the lower chambers containing 500 µL medium with 20% fetal bovine serum and cultured for 24 h. After rinsing with 1× BPS, the invaded cells were stained with .1% crystal violet in PBS and visualized using a microscope. The cell invasion rate was quantified using ImageJ software (National Center for Biotechnology Information).

### 2.5 Quantification of intracellular and extracellular calcium

Intracellular calcium was measured using a Calcein AM, cell‐permeant assay kit (C1430, Invitrogen). Briefly, 10^5^ cells were plated in 12‐well plates and cultured for 24 h. After washing with 1× BPS, a reagent containing rhodamine‐2 (Rhod‐2) was added to the cells and then analyzed under a fluorescence microscope or quantified by a plate reader at 552/581 nm. In addition, Ca^2+^ in the media was measured using a Calcium Colorimetric Assay Kit (MAK022, Sigma‐Aldrich). The media were replaced in the reader plates, and 90 µL of colourimetric reagent was added to 60 µL of assay buffer and incubated for 10 min. The concentration of calcium was measured at 575 nm.

### 2.6 In vitro osteoclastogenesis assay

Osteoclast precursor RAW264.7 cells were stimulated by coculturing with PC‐3 cells under bilayer culture conditions for 24 h, and 10^5^ stimulated cells were replaced in 12‐cell plates. After culturing for 24 h, the cells were incubated with 4% paraformaldehyde for 20 min, and then stained using a tartrate‐resistant acid phosphatase (TRAP) Kit (G1492, Solarbio Life Sciences) for 30 min. After rinsing with 1× BPS, cell nuclei were counterstained with a hematoxylin dye. The relative TRAP density was quantified using a light microscope.

### 2.7 RNA sequencing

RNA was extracted from PCa cells using an RNeasy kit (74104, Qiagen). RNA sequencing was performed by Vazyme Biotech., as previously described.^25^ The mRNA expression profiles were analyzed by the Kyoto Encyclopedia of Genes and Genomes (KEGG) pathway enrichment (https://david.ncifcrf.gov).

### 2.8 Reverse transcription‐quantitative PCR

PCa cell‐extracted RNA was used to synthesize cDNA using a PrimeScript RT Reagent Kit (RR037A, Takara). The cDNA was quantified using SYBR Green PCR Master Mix (409155, Thermo Fisher). The relative S100A4 mRNA level was calculated by normalization to β‐actin mRNA. The PCR primers are shown in Table S1.

### 2.9 Immunofluorescence

PCa cells were seeded onto chamber slides (BD Biosciences) at a density of 2 × 10^3^ and fixed in 4% paraformaldehyde. The cells were permeabilized with .5% Triton X‐100 and then blocked with 5% bovine serum albumin for 30 min. After washing with 1× PBS, the cells were incubated with primary antibodies from Cell Signaling Technology, including AR (54653), RelB (4954), NFATc1 (8032), and S100A4 (13018) antibodies at 4°C for 12 h, and then incubated with Alexa Fluor 647 secondary antibody (ab150115, Abcam) for 1 h. The fluorescence image was captured under a confocal microscope with DAPI counterstaining (Zeiss).

### 2.10 Immunoblots

Total protein and nuclear protein were extracted from tissues and cells using a RIPA lysis buffer (sc‐24948, Santa Cruz Biotechnology) and Nuclear Extraction Kit (40010, Active Motif), respectively. Next, 50–100 µg of extracts were separated on SDS‐PAGE, transferred to PVDF membranes, and then incubated with the primary antibodies at 4°C for 12 h. Except a β‐actin antibody (sc‐47778, Santa Cruz Biotechnology), all antibodies were provided by Cell Signaling Technology, including AR (54653), Ikkα (2682), RelA (9609), RelB (4954), c‐Rel (4727), p105/p50 (3035), p100/p52 (4882), IL‐8 (9447), Sp1 (5931), NFATc1 (8032), Snail 1 (3879), Twist 1 (46702), vimentin (5741), E‐cadherin (3195), N‐cadherin (13116), S100A4 (13018), and PCNA (13110). After washing with TBST buffer, the membranes were incubated with goat an anti‐mouse (or anti‐rabbit) secondary antibody (sc‐362267, sc‐3739, Santa Cruz Biotechnology) for 2 h. The signals were imaged by a chemiluminescence system (Bio‐Rad) and quantified by normalizing with β‐actin and PCNA using Quantity One software.

### 2.11 Immunohistochemical analysis

Tissues from patients and mice were fixed in paraffin‐embedded slides and then incubated with primary antibodies at 4°C for 12 h, including RelB (4954, Cell Signaling Technology), Snail 1 (13099‐1‐AP, Proteintech), Twist 1 (MAB6230, R&D Systems), vimentin (5741, Cell Signaling Technology), and S100A4 (ab218512, Abcam). Next, the tissue slices were reacted with biotinylated a goat anti‐mouse (or anti‐rabbit) secondary antibody (ab6788, ab64256, Abcam) for 30 min. The immunosignal was imaged using a DAB Substrate Kit (8059, Cell Signaling Technology) and quantified using ImageJ software. The immunohistochemical (IHC) intensity was calculated as previously described.^25^

### 2.12 Luciferase reporter response

The transcriptional regulation was estimated using the luciferase reporter expression response. The NF‐κB enhancer and NF‐κB/NFAT super‐enhancer regions were cloned into a pGL4 vector (E6651, Promega) to activate the luciferase reporter. The enhancer function was validated by mutating NF‐κB and NFAT binding sites using a GeneArt site‐directed mutagenesis system (A13282, Thermo Fisher Scientific) with custom‐designed primer sets shown in Table S1. After co‐transfecting the reporter constructs with a β‐galactosidase (β‐gal) cDNA construct into PCa cells, the reporter activity was quantified by Luciferase Assay System (E610, Promega). β‐gal activity was measured using a relative kit (K145501, Thermo Fisher Scientific). The enhancer activity was quantified using β‐gal‐normalized luciferase activity.

### 2.13 Interaction of transcription factors

Sp1 and NF‐κB interaction was examined using a Duolink Proximity Ligation Assay (DUO92202‐1KT, Sigma). Briefly, the cells were blocked on 1 cm^2^ glass slides and incubated with primary antibodies against Sp1 (5931, Cell Signaling Technology) and NF‐κB family members (Cell Signaling Technology). A ligase was added to the slides before incubation with and without proximity ligation assay (PLA) probes for 30 min. The probe signal was amplified using polymerase for 100 min, and then incubated with a Duolink PLA mounting medium with DAPI for 15 min. The signals were imaged under a fluorescence microscope (Nikon). The CheckMate mammalian two‐hybrid system (E2440, Promega) was used to verify RelB‐NFAT1c interaction in the S100A4 enhancer. The human RelB ORF was cloned into a CMV‐binding domain plasmid to link GAL4 fusion, and the human NFAT1c ORF was cloned into a CMA‐activation domain plasmid for VP16 fusion. The generated constructs expressing RelB‐GAL4 and NFAT1c‐VP16 were co‐transfected with a luciferase reporter driven by GAL4‐TATA. Luciferase activity was quantified, and the reporter response was estimated by normalizing to β‐gal activity.

### 2.14 Chromatin immunoprecipitation

Sp1, NF‐κB, and NFAT binding sites were examined using a ChIP‐IT Kit (53008, Active Motif). Chromatin isolated from PCa cells was pulled down by a RelB antibody (ab154957, Abcam). Chromatin without pulldown served as an input control, and pulldown with a rabbit IgG (ab97023, Abcam) served as a negative control. The precipitated enhancer fragments were amplified by PCR and normalized with the input control. The pulldown proteins were also quantified by immunoblotting. The specific ChIP‐PCR primers are shown in Table S1.

### 2.15 Mouse orthotopic and intraskeletal implantation

For metastatic experiments, 5‐week‐old male BALB/c nude mice were provided by Vital River Lab Animal Tech. PC‐3 cells were labelled with luciferase by infecting them with lentivirus‐expressing firefly luciferase, and then stable cell clones were selected using neomycin. The mice were randomized and anaesthetized using 4% chloral hydrate, and 10^5^ cells in 20 µL of 1× BPS were orthotopically implanted into the prostate lobes of mice using a modified surgical procedure.^26^ One week after injection, the mice were imaged weekly by an in vivo imaging system (IVIS) (PerkinElmer). In addition, the mice were examined by BioSpec 94/30 small animal magnetic resonance imaging (Bruker). After 3 weeks, mice were euthanized to examine prostate and metastasized tumours.

RelB was silenced in PC‐3 cells and selected by puromycin. Next, 10^5^ cells in 20 µL of 1× PBS were directly injected into the left intra‐tibias of the mice using 30½ G syringe needles following a previous procedure.^27^ Bone osteolysis in the mice was assessed by X‐ray imaging using an MX‐20 (Faxitron). In addition, S100A4 was silenced in murine PCa RM‐1 cells or ectopically expressed in RelB‐silenced RM‐1 cells. Next, 10^5^ RM‐1 cells in 20 µL of PBS were implanted in caudal arteries using 29 G syringe needles to assess the effect of S100A4 on osteolytic metastasis.^28^ Mice were euthanized to excise the tibias for pathologic examination.

### 2.16 Bone tissue pathological analysis

After staining with hematoxylin and eosin, the bone tissue slices were evaluated under an optical microscope (Olympus). In addition, the tissue slices were stained with a TRAP at 37°C for 2 h and soaked in a hematoxylin dye for 2 min. After washing three times, the dehydrated slices were imaged under a microscope. The TRAP activity was quantified at 400 nm using a multifunction microplate reader (Tecan). Bone density was quantified by imaging using a SkyScan 1176 (Bruker), and the three‐dimensional reconstruction of bone tissues was analyzed using CTvox software. The bone density was quantified using CTan software, and the attenuation coefficient was calculated using the bone mineral density normalized to the density of the standard bone substance. For osteolytic analysis, five parameters were examined: the size of the tumour tissue, the density of the tumour cells, cell division, changes in cellular morphology, and osteolytic lesions. Grades 0–4 were included for each item: normal (0), slight (1), light (2), intermediate (3), and severe (4). The maximum morphological score was 20.

### 2.17 PCa patients

Serum and tumour tissue specimens were collected from PCa patients according to the approved clinical research protocol with written informed consent obtained from the patients. Tumour tissues from patients with Gleason scores of 5–9 (six cases in each group) were selected to examine the expression levels of S100A4 using IHC analysis. The patient's blood samples were spun at 3500 g to collect serum and stored at –80°C. ELISA kits were used to measure PSA (DKK300, R&D Systems), IL‐8 (D8000C, R&D Systems), and S100A4 (ab283547, Abcam) concentrations in the serum specimens. In addition, the corrections of PSA, IL‐8, and S100A4 with patient survival were analyzed using the Kaplan–Meier statistic test. The patient median follow‐up time was 61 months, and the average survival time was 83.1 ± 6 months. The patients were categorized into high‐ and low‐expression groups based on the median expression levels of PSA (22.56 ng/mL), IL‐8 (75 pg/mL), and S100A4 (9.02 ng/mL).

### 2.18 Statistical analysis

The data from at least three replicates are shown as the mean ± standard deviation (SD). Unpaired Student's *t*‐test and one‐way analysis of variance (ANOVA) were used to analyze the significant differences between different groups. Prism (GraphPad) was used to analyze multiple comparisons by setting significance at *p* < .05.

## 3 RESULTS

### 3.1 RelB suppresses AR and activates IL‐8 in PCa progression

In contrast to the role of the canonical NF‐κB pathway in cancer progression,^29^, ^30^ information on the noncanonical NF‐κB pathway in human malignancies is lacking. Hence, we analyzed Oncomine datasets to examine the correlation of RelA or RelB with PCa. RelB was more strongly associated with PCa than RelA (Figure S1A). We also analyzed TCGA datasets to examine the correlation between AR and NF‐κB in PCa cohorts. The results indicated that RelB was negatively associated with AR, but RelA was not (Figure S1B). Correspondingly, we recently reported that the nuclear level of RelB was associated with the Gleason score of PCa patients.^25^ In addition, AR‐null PCa cell lines expressed higher levels of NF‐κB family members than AR‐positive PCa and normal prostate cell lines (Figure S1C), and the nuclear level of RelB was also high in AR‐null PCa cell lines (Figure S1D).

Tumour necrosis factor (TNF)‐α activates PCa cell inflammation without amplifying AR‐regulated genes, suggesting that NF‐κB may functionally compensate for the AR in PCa malignancies.^31^, ^32^ Consistently, TNF‐α‐induced RelB nuclear translocation but suppressed AR nuclear translocation (Figure S1E). In addition, RelB suppressed the AR‐driven luciferase reporter response (Figure S1F–H). Collectively, these results suggest that RelB may contribute to PCa progression after AR functional decline.

IL‐8, a cell‐secreted proinflammatory chemokine, is regulated by NF‐κB and contributes to malignant development.^33^, ^34^, ^35^ We previously showed that the overexpression of RelB in AR‐positive PCa cells increased IL‐8 but decreased PSA.^20^ Correspondingly, TCGA dataset analysis indicated that IL‐8 was more correlated with RelB than RelA in PCa cohorts (Figure S2A). In addition, AR‐null PCa cells expressed more IL‐8 than AR‐positive PCa cells (Figure S2B). PTL, an NF‐κB inhibitor,^36^ suppressed IL‐8 expression in PC‐3 cells mainly by repressing RelB (Figure S2C,D). We constructed a *CXCL8* promoter‐driven luciferase reporter expression to examine whether RelB regulated IL‐8 expression (Figure S2E). As expected, increasing RelB upregulated the reporter response in AR‐positive LNCaP cells and vice versa, and silencing RelB downregulated the reporter response in AR‐null PC‐3 cells (Figure S2F,G). Furthermore, a RelB antibody pulled down a *CXCL8* promoter fragment from PC‐3 cell‐derived chromatin, but the amount of precipitate was decreased by silencing RelB (Figure S2H). These results suggest that IL‐8 may participate in RelB‐promoted PCa progression.

**FIGURE 1:**
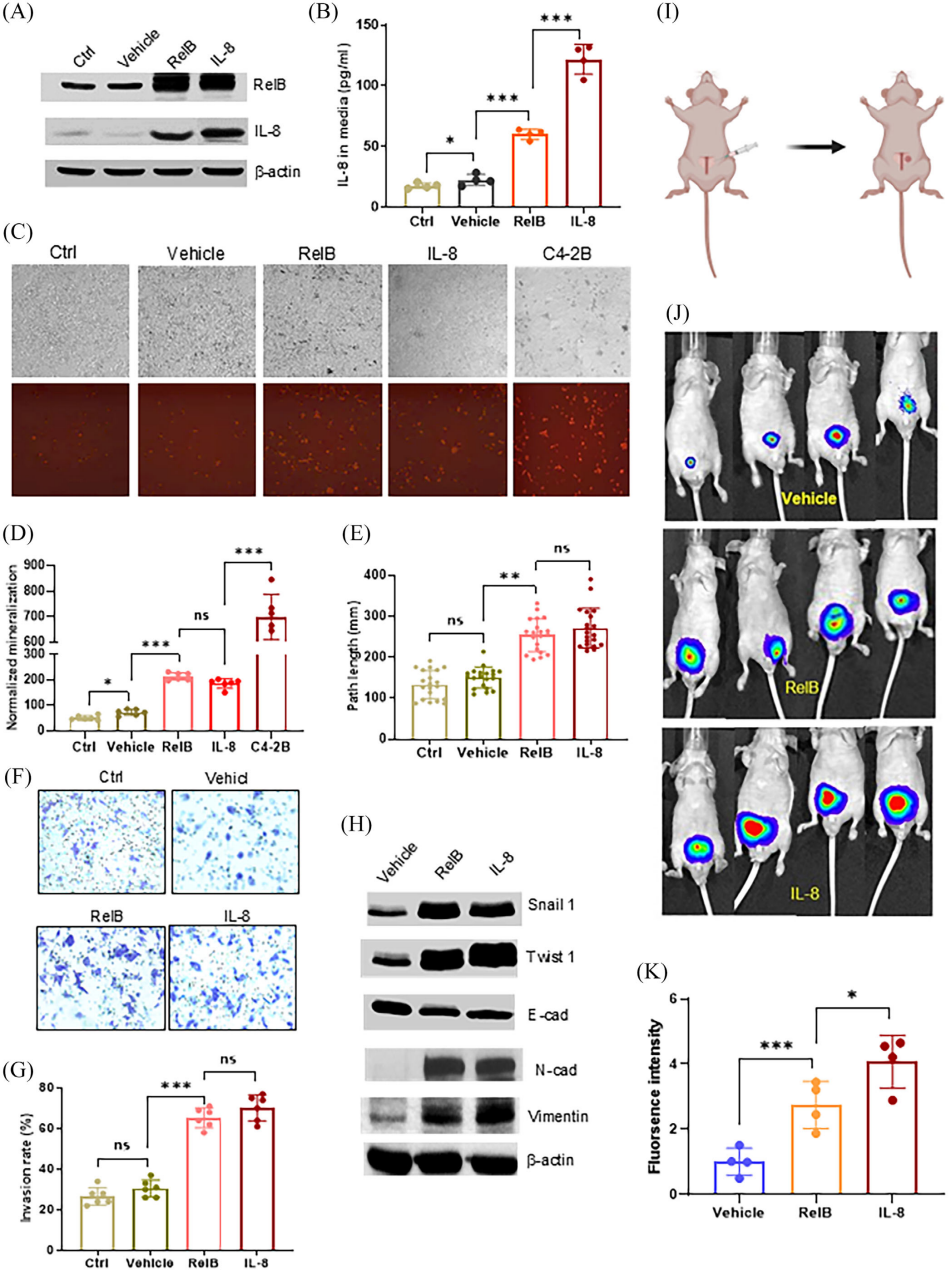
RelB and IL‐8 reciprocity in promoting PCa expansibility. (A) RelB and IL‐8 were ectopically expressed in LNCaP cells, and no transfection served as a control. (B) The concentrations of IL‐8 in established cell lines were measured using a relative ELISA kit (*n* = 4). (C, D) Cell mineralization in RelB‐ or IL‐8‐overexpressed LNCaP cells was quantified with equal cell numbers (upper panel). C4‐2B cell line, an LNCaP cell‐derived bone metastatic lineage, serves as a positive control (*n* = 6). (E) Cell migration in the established LNCaP cell lines (*n* = 20). (F, G) Cell invasion in the established LNCaP cell lines (n = 6). (H) Expression of EMT‐associated proteins in the established LNCaP cell lines. (I) Scheme of orthotopic implantation of RelB‐ or IL‐8‐overexpressed LNCaP cells into nude mice prostates. (J, K) Formed tumours in mouse prostates were imaged by IVIS (*n* = 4). Data are shown as the mean ± SD. *p < .05, **p < .01, ***p < .001, ns, no significance; one‐way ANOVA.

Consequently, we established LNCaP cell‐derived lineages with high levels of RelB and IL‐8 to assess the effect of IL‐8 on PCa progression. RelB and IL‐8 appeared to be reciprocal, indicating that a feed‐forward inflammatory cycle led to cell aggressiveness (Figure 1A). The overexpression of RelB or IL‐8 increased IL‐8 cell secretion (Figure 1B), and high levels of RelB or IL‐8 enhanced cell mineralization, migration, and invasion (Figure 1C–G). Accordingly, increasing RelB or IL‐8 increased levels of N‐cadherin, Snail 1, Twist 1, and vimentin, except E‐cadherin decreased (Figure 1H). The cells were orthotopically implanted into nude mice prostates, and tumour formation was tracked using an IVIS (Figure 1I). As shown in Figure 1J,K, cells with high levels of RelB or IL‐8 fostered tumour growth and dissemination in the mice. However, pathological analysis did not identify metastasized tissues in the excised bones, regardless of the luciferase signal detected in the left tibia. Thus, although increases in IL‐8 in LNCaP cells strongly affected prostate tumour expansion, the malignant potency of IL‐8 was insufficient to drive bone metastasis (Figure S2I).

### 3.2 RelB cooperates with Sp1 in EMT escalation

**FIGURE 2:**
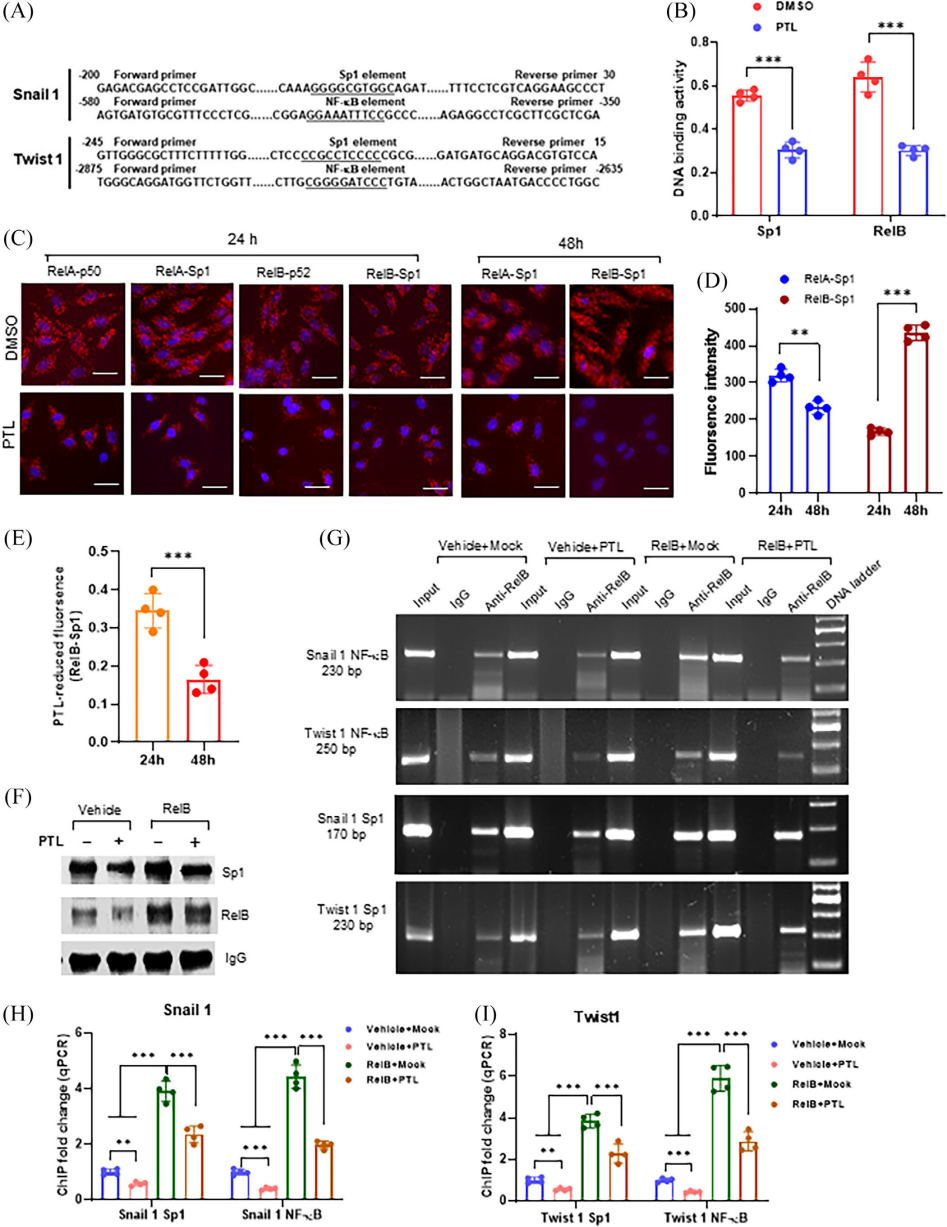
Sp1‐RelB cooperation in regulating *SNAL1* and *TWIST1* genes. (A) Sp1 and NF‐κB binding sites were identified in a 5′ flanking region of the human *Snall 1* and *Twist 1* genes. PCR primer sets were designed to amplify DNA fragments containing the binding sites, the sequence numbers are according to the transcriptional start site as +1. (B) PLT suppressed the Sp1 and NF‐κB binding activity in PC‐3 cells. (C–E) The Sp1 and NF‐κB interaction in PC‐3 cells was analyzed using Duolink PLA assay. The interaction of p50:RelA and p52:RelB dimers served as positive controls, and PLT was used as an inhibiter (C). RelA‐Sp1 and RelB‐Sp1 interactions were determined at 24 and 48 h (D). PLT inhibited the RelB‐Sp1 interaction (E). (F‐I) The Sp1 and NF‐κB binding sites were precipitated from chromatins derived from RelB‐overexpressed LNCaP cells by ChIP using a RelB antibody. Chromatins without pulldown served as input controls, pulldown with IgG served as negative antibody controls, and PLT served as an inhibiter. Sp1 and RelB proteins in the precipitated complexes were quantified by immunoblotting using specific antibodies, and IgG served as a loading control (F). Precipitated Sp1 and NF‐κB binding sites were amplified by regular PCR (G) and qPCR (H, I) with specific primers shown in (A) and Table S1. Data are shown as the mean ± SD. *n* = 4, **p < .01, ***p < .001; *t*‐text (B, D, E), one‐way ANOVA (H, I). Scale bar, 20 µm in (C).

We identified the Sp1 and NF‐κB binding sites located in the promoter and enhancer regions of the *SNAI1* and *TWIST1* genes, respectively (Figure 2A). PTL repressed both DNA‐binding activities in PC‐3 cells (Figure 2B). Sp1 and NF‐κB cooperation in transcriptional regulation was assessed using a dual‐link system compared with RelA:p50 and RelB:p52 dimers as positive controls. Sp1‐RelA interaction occurred at 24 h, and Sp1‐RelB interaction stepped up at 48 h. PTL was sufficient to counteract the Sp1‐RelB interaction (Figure 2C–E). In addition, we performed a ChIP assay to quantify the Sp1‐RelB interaction in regulating the *SNAI1* and *TWIST1* genes. Expectedly, the RelB antibody precipitated both RelB and Sp1 proteins in chromatin isolated from RelB‐overexpressing LNCaP cells (Figure 2F). The RelB antibody not only pulled down NF‐κB enhancer fragments but also precipitated Sp1 promoter fragments from both the *SNAI1* and *TWIST1* genes (Figure 2G). Elevations in RelB led to increased precipitated fragments; however, PTL treatment efficiently alleviated the RelB effect (Figure 2H,I). We also silenced Snail 1 and Twist 1 in RelB‐overexpressing LNCaP cells against RelB function in EMT. As expected, the silence of Snail1 and Twist1 decreased N‐cadherin and vimentin, except E‐cadherin increased (Figure S3A). Correspondingly, the deprivation of Snail 1 and Twist 1 impeded cell migration and invasion by counteracting RelB function (Figure S3B–D), suggesting that RelB enhances EMT mainly through Snail and Twist activation.

### 3.3 S100A4 enhances PCa aggressiveness via EMT activation

**FIGURE 3:**
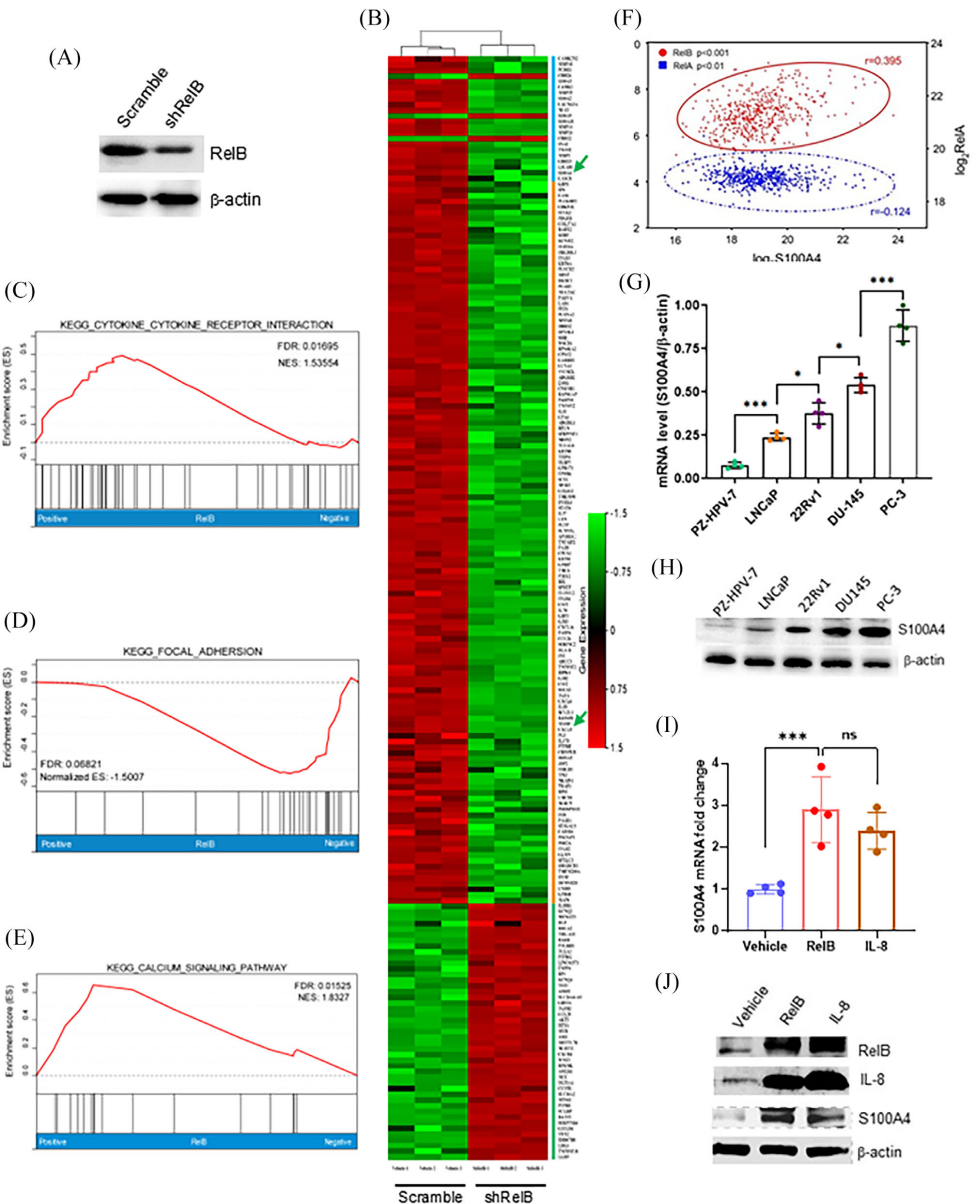
The mRNA expression profiles in RelB‐silenced PC‐3 cells. (A, B) After silencing RelB in PC‐3 cells (A), the mRNA expression profiles were examined by RNA sequencing. IL‐8 (CXCL8) and S100A4 are indicated by green arrows (B). (C–E) The mRNA profiles were analyzed by KEGG pathway enrichment, assembling in cytokine (C), cell adhesion (D), and calcium signalling (E). (F) The correlation between S100A4 and RelB or RelA in PCa was analyzed using the Oncomine database. (G, H) S100A4 mRNA and protein levels in PCa cell lines vs. a normal cell line (PZ‐HPV‐7). (I, J) S100A4 mRNA and protein levels in RelB‐ or IL‐8‐overexpressed LNCaP cells. Data are shown as the mean ± SD. *n* = 4, *p < .05, ***p < .001, ns, no significance; one‐way ANOVA).

Castration treatment frequently promotes PCa recurrence in patients with mCRPC subtypes.^37^, ^38^ RelB‐regulated genes relevant to PCa metastasis were identified by analyzing the mRNA profiles in RelB‐deprived PC‐3 using RNA‐seq (Figure 3A,B). Data assessment of enhanced KEGG signalling pathways revealed that RelB was essential for regulating multiple signalling pathways, including cytokines, cell adhesion, and calcium metabolism (Figure 3C–E). Notably, members of the calcium‐binding S100A family were downregulated in RelB‐silenced cells, particularly S100A4, which is relevant to malignancies^23^ (Table 1). Consistently, the Oncomine dataset analysis indicated that S100A4 was correlated with RelB in PCa progression, but not RelA (Figure 3F). S100A4 was highly expressed in AR‐null PCa cells compared with AR‐positive PCa cells (Figure 3G,H), which was correlated with RelB and IL‐8 levels (Figure 3I,J).

**TABLE 1:** mRNA expression profiles (shRelB vs. shCtrl).

Gene ID	Log_2_ fold change	*p*‐value
Cytokines/chemokines		
IL‐6	−2.26	.011797
IL‐8	−1.20	.001405
CXCL1	−5.24	.000198
CXCL16	−1.57	.003708
IL‐1B	−2.03	.000244
IL‐11	−2.94	.000709
IL‐17	−1.32	.019744
EMT associated factors		
SNAI	−1.55	.00027
TWIST	−1.45	.00046
VIM	−1.76	.0003
CDH12	2.05	.00027
CDH26	3.43	.00837
CDH13	−7.68	.00027
Calcium metabolism factors		
NFAT	−2.86	.00027
S100A2	−1.66	.00027
S100A3	−2.03	.00049
S100A4	−2.40	.00027
S100A9	1.34	.00027
S100A10	−1.17	.00034
MMP1	−1.50	.00027
MMP14	−1.04	.00073
MMP15	−1.01	.00027
CAMK2N2	−1.46	.00665
CACNA1A	−1.53	.00027

**FIGURE 4:**
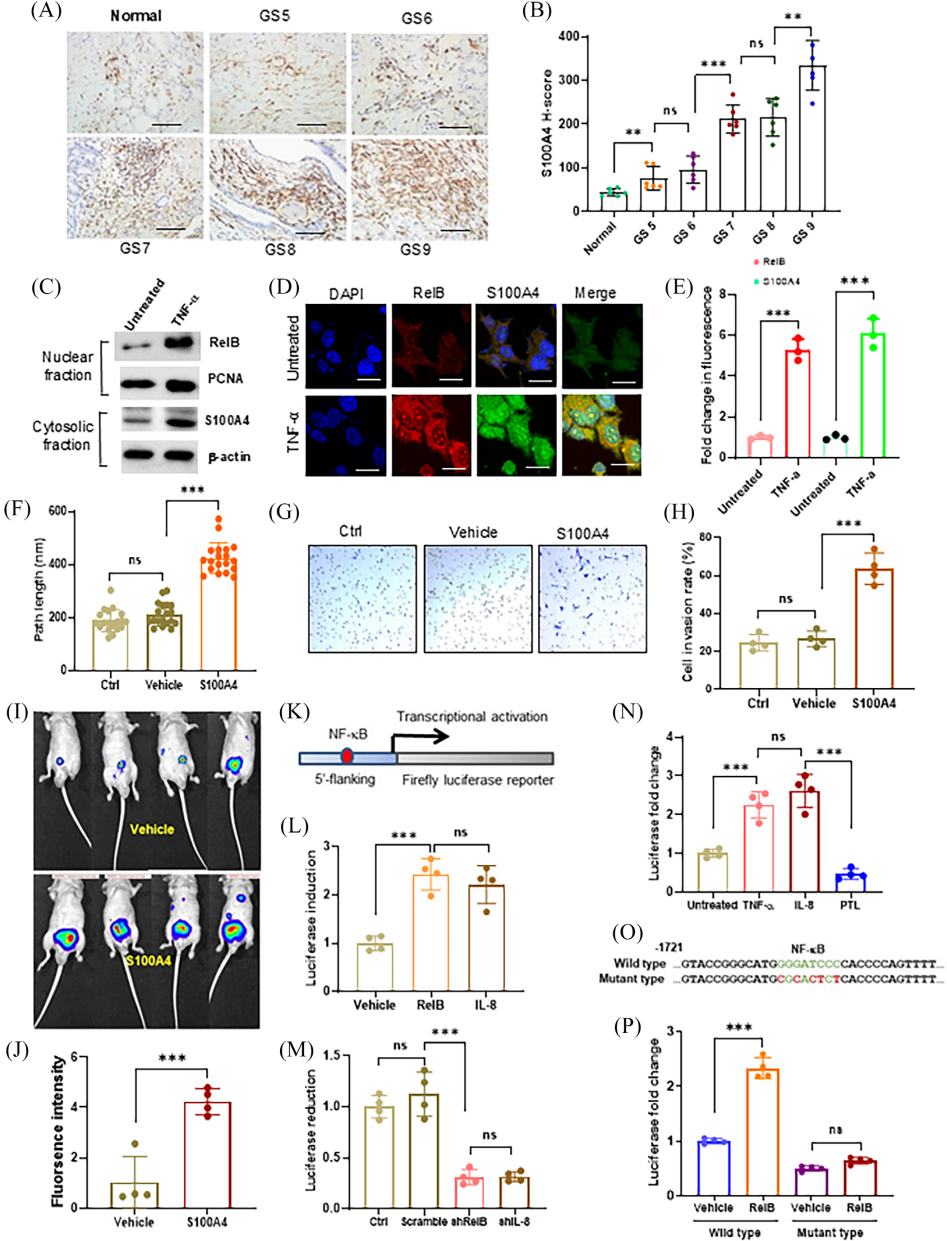
RelB‐upregulated S100A4 in promoting PCa aggressiveness. (A, B) The correlation of S100A4 with Gleason score (GS) of PCa patients (*n* = 6). (C–E) TNF‐α increased the levels of nuclear RelB and cytosolic S100A4 in LNCaP cells. (F) The effect of S100A4 on LNCaP cell migration (*n* = 4). (G, H) The effect of S100A4 on LNCaP cell invasion (*n* = 4). (I, J) The S100A4‐overexpressed LNCaP cells were orthotopically implanted into nude mice prostates to examine the effect of S100A4 on tumour formation (*n* = 4). (K) The S100A4 enhancer was linked to the luciferase reporter. (L‐N) The reporter responses in RelB‐ and IL‐8‐overexpressed LNCaP cells (L), RelB‐ and IL‐8‐silenced PC‐3 cells (M), and TNF‐α‐ and PTL‐treated PC‐3 cells (N) (*n* = 4). (O, P) The RelB‐binding site was mutated to eliminate the reporter response in PC‐3 cells (*n* = 4). Data are shown as the mean ± SD. **p < .01, ***p < .001, ns, no significance; *t*‐test (E, J), one‐way ANOVA (B, F, H, L–N, P). Scale bar, 50 µm in (A) and 20 µm in (D).

Patient tumour tissues were analyzed by IHC assays using an S100A4 antibody. The results confirmed that constitutive S100A4 was associated with patient Gleason scores (Figure 4A,B). In addition, TNF‐α induced the expression of S100A4 by increasing RelB nuclear translocation (Figure 4C–E). S100A4 was ectopically expressed in LNCaP cells to determine whether S100A4 promoted PCa metastasis (Figure S4A). As expected, increasing S100A4 enhanced cell migration and invasion (Figure 4F–H). Consequently, we orthotopically implanted the cells into mice prostates, and the results confirmed that high levels of S100A4 significantly promoted tumour expansion (Figure 4I,J). Correspondingly, we identified an NF‐κB binding site in a 5′‐flanking region of the *S100A4* gene, which was responsive to RelB‐ or IL‐8‐mediated transcriptional activation in LNCaP cells (Figure 4K,L). Conversely, silencing RelB or IL‐8 led to transcriptional repression in PC‐3 cells (Figure 4M). TNF‐α and IL‐8 stimulated, but PLT repressed the RelB‐activated reporter response (Figure 4N). Consequently, mutating the unique NF‐κB binding site sufficiently abolished RelB‐mediated transcriptional activation (Figure 4O,P).

### 3.4 RelB cooperates with NFAT1c in upregulating S100A4

Interestingly, we identified an NFAT binding site next to the NF‐κB binding site in the *S100A4* enhancer region, which is a transcription factor activated by phosphatase calcineurin and plays crucial roles in T‐cell function and tumour immune evasion.^39^, ^40^ Accordingly, increasing NFATc1 expression upregulated S100A4 expression in LNCaP cells (Figure S4B–D). By contrast, silencing NFATc1 decreased S100A4 expression in PC‐3 cells (Figure S4E,F). Furthermore, PMA induced, but FK506 suppressed S100A4 expression by modulating NFATc1 nuclear levels (Figure S4G–K). Thus, mutating the NFAT binding site could eliminate the NFATc1 enhancer response, suggesting that NFATc1 participates in the transcriptional regulation of the *S100A4* gene (Figure S4L,M).

**FIGURE 5:**
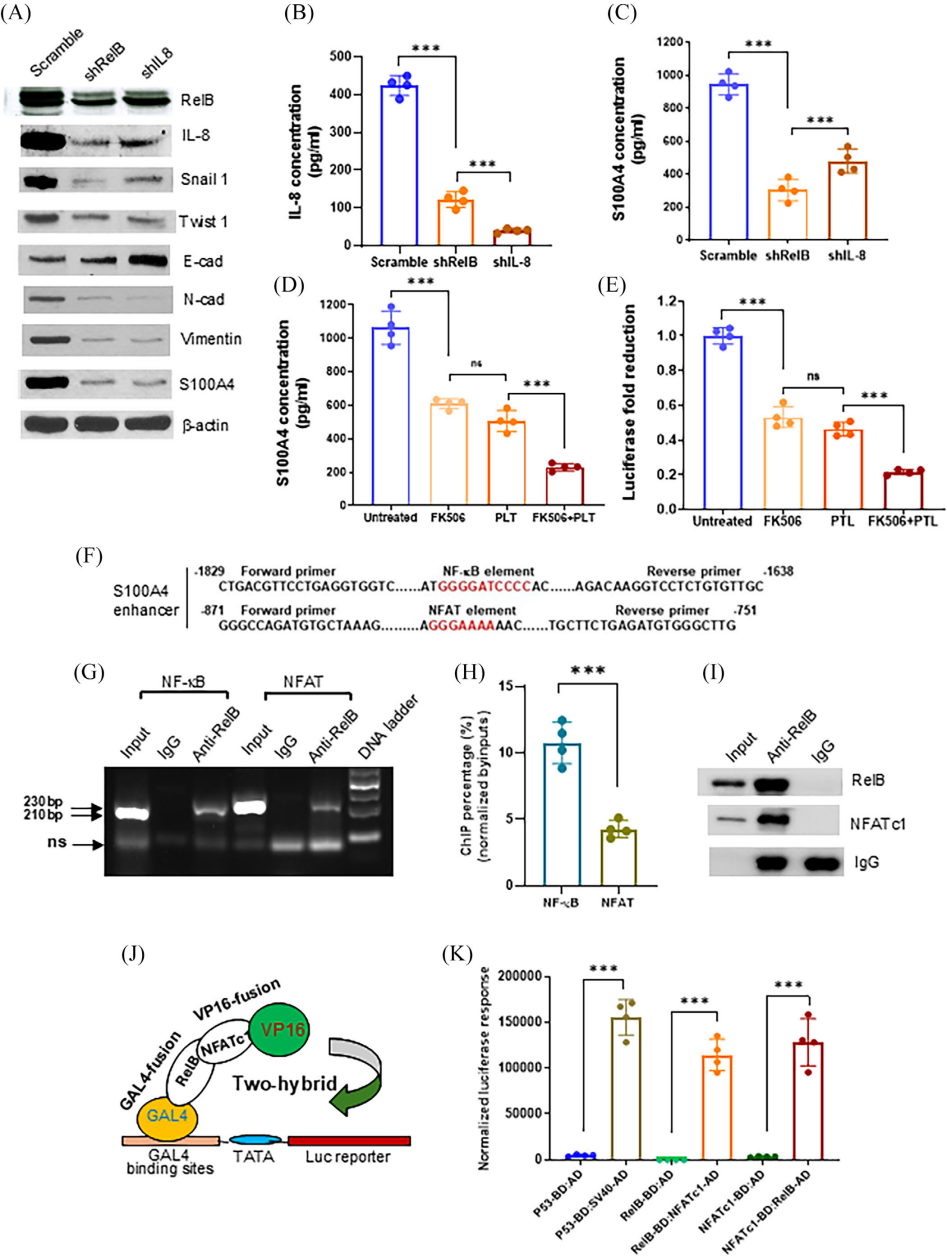
RelB and NFATc1 cooperation in upregulating S100A4. (A) RelB and IL‐8 were silenced in PC‐3 cells, and the expression levels of S100A4 and EMT‐associated proteins were quantified. (B, C) The levels of IL‐8 and S100A4 in PC‐3 cell culture media (*n* = 4). (D) The levels of S100A4 in PTL‐ and FK506‐treated PC‐3 cells (*n* = 4). (E, F) PTL and FK506 inhibited the *S100A4* enhancer‐driven reporter response in PC‐3 cells (*n* = 4). (G–I) The NF‐κB‐ and NFAT‐binding sites in the S100A4 enhancer were precipitated from PC‐3 cell‐derived chromatins by ChIP using a RelB antibody and amplified by regular PCR (G) or qPCR (H). RelB and NFATc1 proteins in the precipitated complexes were quantified by immunoblotting. (I). (J) The RelB‐NFATc1 interaction was determined using a two‐hybrid system as indicated. (K) The integration of RelB and NFATc1 was examined by luciferase response driven by conjugating the VA16‐activation domain (AD) and the GAL4‐binding domain (BD). P53‐BD/VA40‐AD served as the positive control (n = 4). Data are shown as the mean ± SD. ***p < .001, ns, no significance; *t*‐test (H, K), one‐way ANOVA (B–E).

We further elucidated the molecular mechanism by which the NF‐κB/NFAT super‐enhancer regulates S100A4 expression in PCa cells. The silence of RelB or IL‐8 in PC‐3 cells decreased cell‐secreted IL‐8 and S100A4 levels, thereby affecting EMT‐associated protein levels (Figure 5A–C). Consistently, silencing RelB and IL‐8 reduced cell migration and invasion (Figure S5A–C). As a metastatic PCa cell line, PC‐3 is unable to form mineralization in culture; however, silencing RelB and IL‐8 slightly increased cell mineralization (Figure S5D,E). PLT and FK506 treatment reduced S100A4 expression by repressing RelB/NFAT enhancer activity (Figure 5D,E). In addition, we precipitated PC‐3 cell‐derived chromatin using a RelB antibody and quantified the NF‐κB and NFAT enhancer fragments by PCR analysis. The antibody not only precipitated the NF‐κB fragment but also pulled down the NFAT fragment and their bound proteins, indicating that RelB and NFATc1 proteins existed in an enhancer complex (Figure 5F–I). Furthermore, the results of the GAL4‐VP16 two‐hybridization analysis confirmed the functional conjugation of RelB‐NFATc1 in regulating the reporter response (Figure 5J,K). The results suggest that the NF‐κB/NFAT super‐enhancer promoted S100A4 expression in response to increased inflammation.

### 3.5 RelB‐activated S100A4 fosters osteolytic metastasis

**FIGURE 6:**
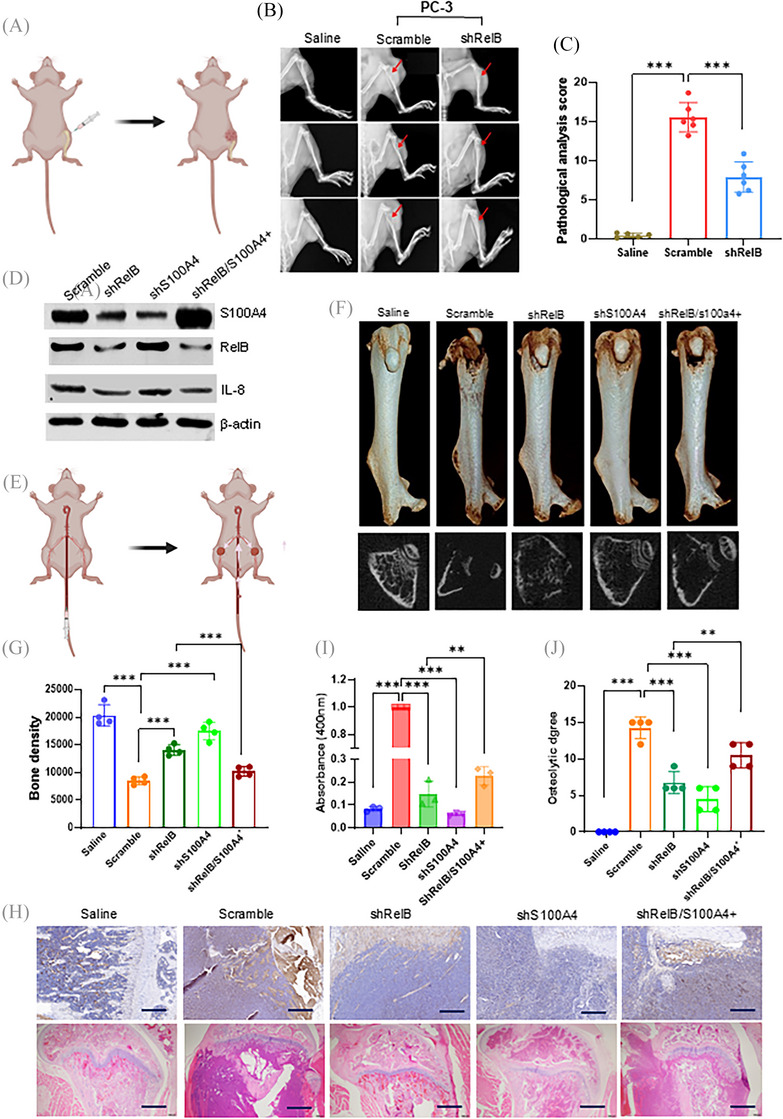
The effects of RelB and S100A4 on osteolytic metastasis. (A) Scheme of intraskeletal implantation of RelB‐silenced PC‐3 cells in nude mice. (B, C) The effect of RelB on osteolytic lesions was examined, and osteolytic lesions were indicated by red arrows. Saline injection served as a control (*n* = 6). (D) RelB and S100A4 were manipulated in RM‐1 cells for bone metastasis in mice and confirmed by immunoblotting. (E) Scheme of caudal arterial implantation of RM‐1 cells into nude mice. (F, G) Osteolytic lesions and bone density in the tibia were examined (*n* = 4). (H–J) Bone metastasis and damage were analyzed using TRAP assay, and H&E (H), Bone metastasis (I), and bone loss (J) were quantified. Data are shown as the mean ± SD. **p < .01, ***p < .001; one‐way ANOVA. Scale bar, 50 µm in (H).

As a crucial calcium‐binding protein, S100A4 increases osteolytic lesions by promoting osteoclastogenesis.^22^, ^23^ We first examined the effect of S100A4 on EMT and calcium homeostasis. As expected, increasing S100A4 enhanced cell mobility by influencing the expression of EMT‐relative proteins in LNCaP cells (Figure S6A,B). Notably, elevating RelB, NFARc1, and S100A4 apparently changed calcium homeostasis by increasing the calcium concentration in cells but decreasing it in the media (Figure S6C–E). By contrast, silencing S100A4 in PC‐3 cells decreased cell mobility by inhibiting EMT (Figure S6F,G). The suppression of RelB, NFARc1, or S100A4 increased calcium consumption in PC‐3 cells and released calcium into the media (Figure S6H,I). RelB and S100A4 functions in osteoclastogenesis were examined in PC‐3 cell‐stimulated osteoclast precursor RAW264.7 cells. The silencing RelB or S100A4 in PC‐3 cells decreased TRAP levels in RAW264.7 cells, but restoring S100A4 reversed the effect of RelB deprivation (Figure S7A). RelB‐depleted PC‐3 cells were intrathecally implanted into the knee articulations of the mice to determine whether RelB was essential for enhancing osteolytic lesions. Although the injection of PC‐3 cells successfully generated osteolytic lesions, RelB deprivation in the cells alleviated bone loss (Figure 6A–C). The bone tissue IHC images indicated that silencing RelB significantly decreased Snail 1, Twist 1, and S100A4 levels in parallel (Figure S7B).

We also manipulated the levels of RelB and S100A4 in murine PCa RM‐1 cells to examine bone metastasis in mice by injecting the cells into tail arteries using a procedure developed by Kuchimaru and colleagues^28^ (Figure 6D,E). After 3 weeks, we euthanized the mice to examine metastatic bone lesions. RM‐1 cells efficiently metastasized to the bone, and bone density was reduced compared with the saline injection controls. The silencing of RelB or S100A4 in RM‐1 cells efficiently reduced bone damage, but restoring S100A4 in RelB‐depleted cells caused a relapse of osteolytic lesions, indicating that increasing S100A4 could counteract the effect of RelB deprivation (Figure 6F,G). IHC imaging indicated that Snail1, Twist1, and vimentin were decreased in groups injected with RelB‐ or S100A4‐silenced cells but further restored as S100A4 was recovered (Figure S7C). In addition, the TRAP assay showed that injected RM‐1 cells formed osteolytic lesions, which were protected by silencing RelB or S100A4, but S100A4 recovery reproduced osteolytic lesions (Figure 6H,I). Consistently, X‐ray imaging confirmed reductions in bone density by bone loss as the tumour increased, but reducing S100A4 unequivocally protected against net bone loss by alleviating the metastatic capacity of RelB (Figure 6H,J).

### 3.6 IL‐8 and S100A4 correlate with PCa osteolytic metastasis

**FIGURE 7:**
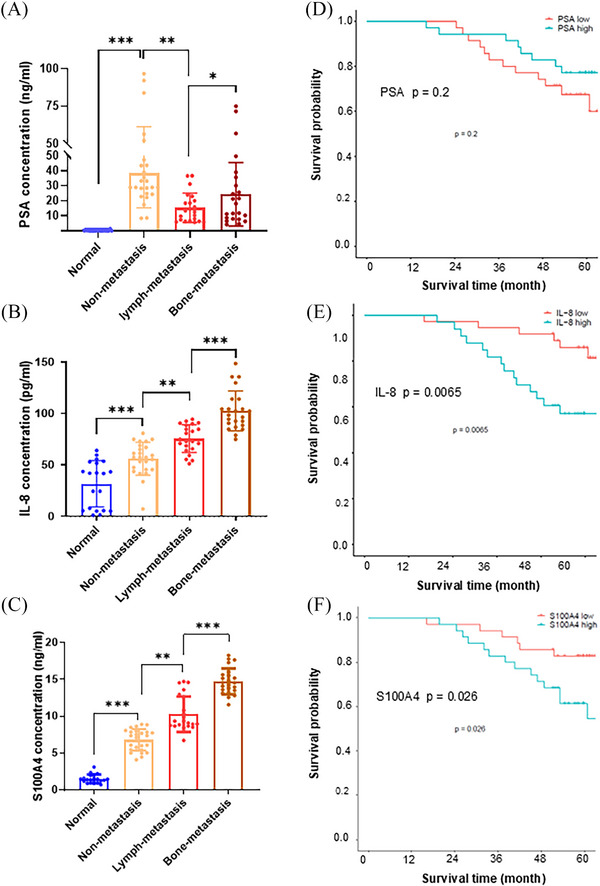
The correlations of PSA, IL‐8, and S100A4 to PCa patients’ malignancy. (A–C) Concentrations of PSA (A), IL‐8 (B), and S100A4 (C) in serum specimens from PCa patients (*n* = 70) vs. normal healthy donors (*n* = 20). (D–F) The corrections of PSA (D), IL‐8 (E), and S100A4 (F) with patients’ survival. Samples from PCa patients included 25 non‐metastasis, 22 lymph‐metastasis, and 23 bone‐metastasis). Data are shown as the mean ± SD. *p < .05, **p < .01, ***p < .001; one‐way ANOVA.

IL‐8 and S100A4 are small cell‐secreted proteins that can be quantified in tissues and the circulatory system.^41^, ^42^ Their potential as useful biomarkers for predicting PCa bone metastasis was assessed by quantifying PSA, IL‐8, and S100A4 in sera from selected PCa patients (Table 2). Serum PSA levels were high in patients with primary prostate tumours compared with healthy donors; however, they decreased in patients with lymph metastasis and further increased in patients with bone metastasis (Figure 7A). The serum levels of IL‐8 and S100A4 steadily increased following stage‐by‐stage PCa metastatic progression, suggesting that IL‐8 and S100A4 may serve as metastatic PCa biomarkers beyond PSA (Figure 7B,C). In addition, the high levels of IL‐8 and S100A4 were correlated to poor survival compared to PSA levels with no significant effect on patient survival (Figure 7D–F). Overall, the results from this study revealed that the RelB‐IL‐8‐S100A4 axis orchestrates osteolytic lesions via transcriptional scaffolds. Accordingly, the relative DNA binding sites of NF‐κB and its co‐factors in the RelB‐regulated genes are listed in Figure S8A–C, and the mutated NF‐κB and NFAT binding sites in the human *S100A4* gene were indicated in Figure S8D,E.

**TABLE 2:** Quantitative analyses of PSA, IL‐8, and S100A4 in prostate cancer patients’ serum specimens.

Variable	Number of cases	PSA (ng/mL)	IL‐8 (pg/mL)	S100A4 (ng/mL)
Age (years)				
<70	34	25.51	74.28	10.89
≥70	36	27.45	80.58	10.13
Gleason score				
3+3	4	35.00	34.56	6.71
3+4	5	43.74	45.20	7.60
4+3	12	37.47	63.56	6.42
4+4	13	26.56	71.83	8.44
4+5	14	23.04	81.36	13.31
5+4	15	15.16	90.22	13.03
5+5	7	21.73	124.74	14.50
Grade^a^				
1	4	35.00	34.56	6.71
2	5	43.74	45.20	7.60
3	14	33.85	62.02	7.48
4	19	21.65	74.02	9.75
5	28	21.84	99.55	13.57
PSA (ng/mL)				
>4<10	18	6.96	86.05	11.99
>10<30	30	20.38	75.68	10.27
>30	22	50.87	73.04	9.59
Metastasis status				
Nometastasis	25	38.32	56.06	6.70
Lymph metastasis	22	15.33	75.61	10.28
Bone metastasis	23	24.37	102.7	14.74
Normal control^b^	20	.24	31.66	1.50

^a^ According to the new ISUP 2014/WHO 2016 grading system of prostate cancer.

^b^ The experiment included 20 normal serum samples from healthy male donors.

## 4 DISCUSSION

AR‐independent metastatic PCa is resistant to the common anticancer treatments, leading to poor prognosis, particularly from bone metastasis.^1^, ^15^, ^43^ The interplay between ARs and inflammatory signalling plays a crucial role in mCRPC development.^32^, ^44^ However, the functional orchestration by multiple factors is complicated, and its dynamic process needs to be better understood. Although NF‐κB preferentially upregulates AR splicing variants that lack the ligand‐binding domain,^45^, ^46^ NF‐κB promotes the mCRPC phenotype presumably via the negative regulation of the AR cistrome, irrespective of the general reciprocal tethering of AR to NF‐κB on chromatin.^32^, ^47^ In this regard, we previously demonstrated increases in IL‐8 levels as PSA levels declined during PCa progression.^20^ In addition to an earlier finding in Canadian PCa patients with high nuclear RelA levels,^48^ this study revealed that RelB and IL‐8 synergistically fostered AR‐independent proliferation by preventing AR nuclear translocation. This finding suggests that proinflammatory signalling substitutes for AR signalling to sustain PCa progression after AR functional decline.

During metastatic dissemination, cancer cells undergo de‐differentiation processes, such as EMT, to invade the surrounding tissues.^49^, ^50^ Snail and Twist serve as the master transcription factors in regulating EMT, which drives mesenchymal phenotype and promotes cancer metastasis by upregulating genes associated with EMT, including N‐cadherin and vimentin.^51^, ^52^ However, EMT is essential for cancer cell invasion but not rate‐limiting for metastasis.^53^, ^54^, ^55^ Particularly, EMT is insufficient for developing bone metastasis, and the inflammatory signalling and various growth factors contribute to promoting metastatic cells targeting the bone.^56^, ^57^ This study further demonstrated that S100A4 plays a crucial role in prostate cancer osteolytic metastasis, although IL‐8‐activated EMT is necessary to promote cancer expansion. As critical tumour microenvironmental factors, IL‐6, IL‐8, TGF‐β, and TNF‐α, facilitate EMT‐associated metastasis.^33^ The IL‐6‐mediated inflammatory loop enhances cancer metastasis and drug resistance via EMT activation.^58^, ^59^ IL‐8 activation enhances tumour malignancy by inducing EMT.^19^, ^60^ The therapeutic targeting of TGF‐β prevents cancer invasion and metastasis by inhibiting the EMT process.^61^ However, the mechanisms by which inflammatory signalling triggers EMT remain to be fully elucidated. Thus, to explore the upstream cues for Snail/Twist‐activated EMT, this study demonstrated that RelB‐Sp1 cooperation was sufficient for the transcriptional upregulation of Snail1 and Twist1.

Bone metastasis is one of the most common complications of many cancers and is frequent in prostate and breast cancers.^6^, ^62^ The interplay of tumour cells with the bone microenvironment mainly determines bone metastasis by remodelling the bone matrix through delicately balancing osteoblastogenesis and osteoclastogenesis.^63^, ^64^ In this regard, tumour cell‐derived growth factors activate bone morphogenetic proteins and matrix metalloproteinase proteins, which are crucial for promoting osteoblastic lesions,^65^, ^66^ while tumour cell‐derived cytokines stimulate local RANK activation to incite osteoclast‐mediated osteolysis.^8^, ^67^, ^68^ Nevertheless, the mechanisms underlying RANK‐activated osteolytic metastasis remain obscure. To this end, this study revealed that RelB promoted PCa osteolytic metastasis mainly by concerting the effects of IL‐8 and S100A4, which was further validated using multiple mouse orthotopic bone metastasis models.

In summary, this study demonstrated that IL‐8 fosters PCa osteolytic metastasis by coordination with S100A4, and the two malignant factors may serve as a valuable biomarker for predicting PCa osteolytic metastasis. Mechanistically, hyperactivation of RelB transcriptionally upregulated IL‐8 to promote EMT as AR function declined. RelB also upregulated S100A4 to provoke osteolytic metastasis. The findings suggest that blocking the RelB‐IL‐8‐S100A4 axis is a promising approach for preventing osteolytic metastasis.

## AUTHOR CONTRIBUTIONS

Wenbo Sun, Kenny Xu, and Peipei Qian developed the methodology, performed experiments, and analyzed data. Xiao Li, Yanyan Zhang, Fan Xu, Xiaowei Wei, Zhi Xu, and Jiaji Ding acquired and analyzed data. Xiao Li, Xiumei Wang, and Xinyu Xu provided technical and material support. Qin Jiang provided administrative support. Yong Xu conceived the study, interpreted the data, and provided funding support. Kenny Xu and Yong Xu wrote the draft and revision of the manuscript.

## CONFLICT OF INTEREST STATEMENT

The authors declare no conflict of interest.

## ETHICS STATEMENT

Institutional Animal Care and Use approved by the Research Committee of Nanjing Medical University (no. IACUC‐1901031). Patient study protocol approved by the Ethics Committee of Nanjing Medical University (2018‐565).

## Supporting information



Supporting Information

## Data Availability

The TCGA and the Oncomine database are accessible from https://portal.gdc.cancer.gov/ and https://www.oncomine.org/resource. The data are available in a public open‐access repository. Additional information required to reanalyze the data presented in this study is available for reasonable requests to Dr. Yong Xu.
