# Supplementary material for: Insight into prostate cancer osteolytic metastasis by RelB coordination of IL‐8 and S100A4
Source: Clin Transl Med. 2024 Oct 16;14(10):e70058. doi: 10.1002/ctm2.70058 (PMC11483529; doi:10.1002/ctm2.70058)
Supplement: Supplementary file 1 — Supporting Information [file CTM2-14-e70058-s001.pdf]

Figure S1

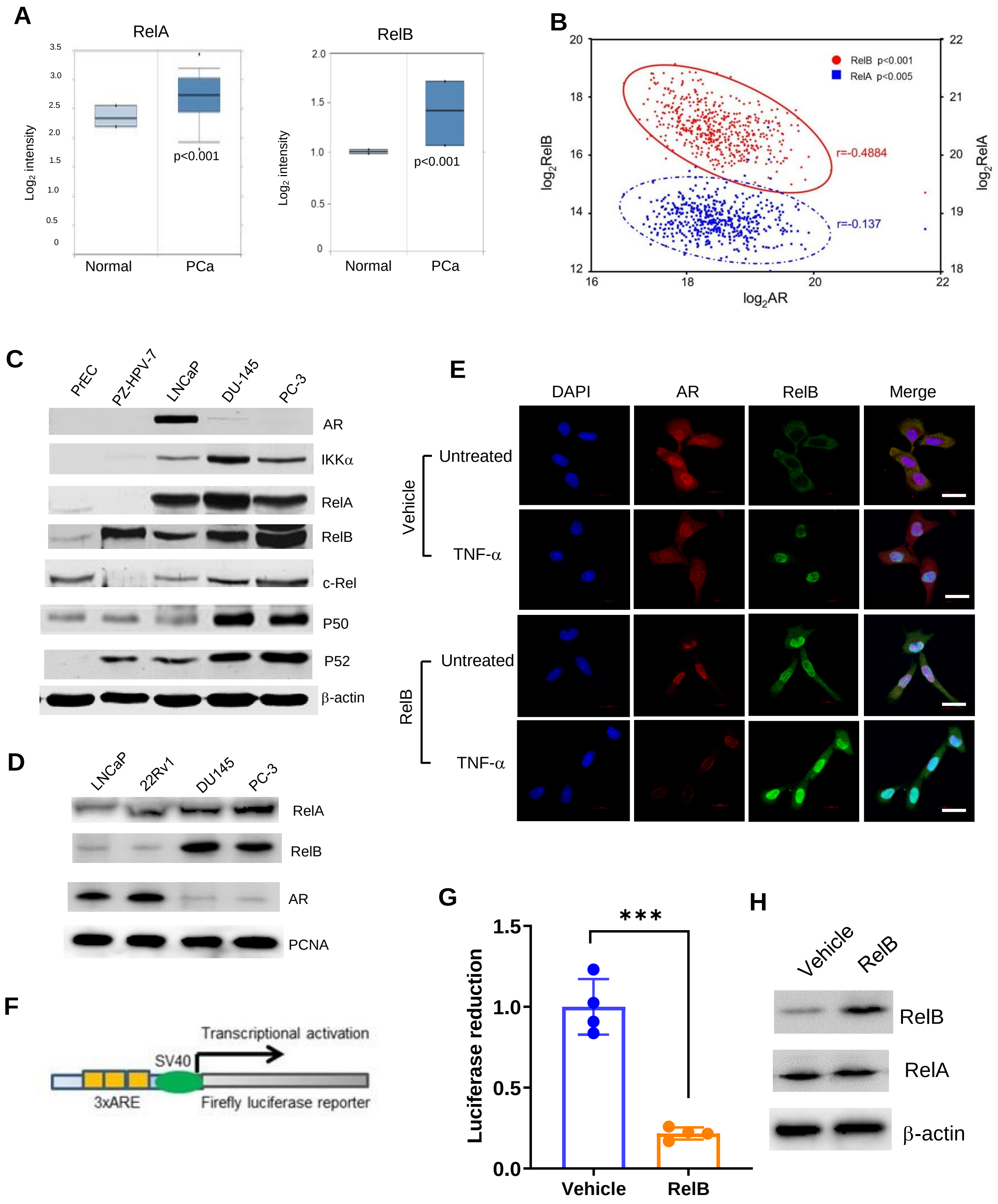
A
RelA
RelB
Log2 intensity
Normal
PCa
p<0.001
B
log2RelB
log2RelA
log2AR
RelB p<0.001
RelA p<0.005
r=-0.4884
r=-0.137
C
PrEC
PZ-HPV-7
LNCaP
DU-145
PC-3
AR
IKKα
RelA
RelB
c-Rel
P50
P52
β-actin
D
LNCaP
22Rv1
DU145
PC-3
RelA
RelB
AR
PCNA
E
DAPI
AR
RelB
Merge
Vehicle
RelB
Untreated
TNF-α
F
Transcriptional activation
SV40
3xARE
Firefly luciferase reporter
G
Luciferase reduction
Vehicle
RelB
***
H
Vehicle
RelB
RelB
RelA
β-actin

Fig. S1. Inverse correlation of RelB and AR in PCa progression.

(**A**) The correlation of RelA or RelB with PCa was assessed using the Oncomine database. (**B**) The correlation of RelA or RelB with AR in PCa tumor tissues was analyzed using the TCGA database. (**C**) The expression levels of NF-κB family members in human AR-null PCa cell lines (PC-3 and DU-145), AR-positive PCa cell line LNCaP, and prostate epithelial cell lines (PrEC and PZ-HIPV-7). (**D**) The levels of nuclear RelA, RelB, and AR in AR-null cell lines (DU-145 and PC-3) vs. AR-positive cell lines (LNCaP and 22Rv1). (**E**) The effect of TNF-α in RelB and AR nuclear translocation in LNCaP cells. (**F-H**) The AR elements (3×ARE) were linked to luciferase reporter as indicated in (F). The effect of RelB on AR-driven transcriptional regulation was estimated by quantifying the reporter responses in LNCaP cells (G, H). Data are shown as the mean ±SD. n=4, ***$p<0.001$, t-test. Scale bars, 20 μm in (E).

Figure S2

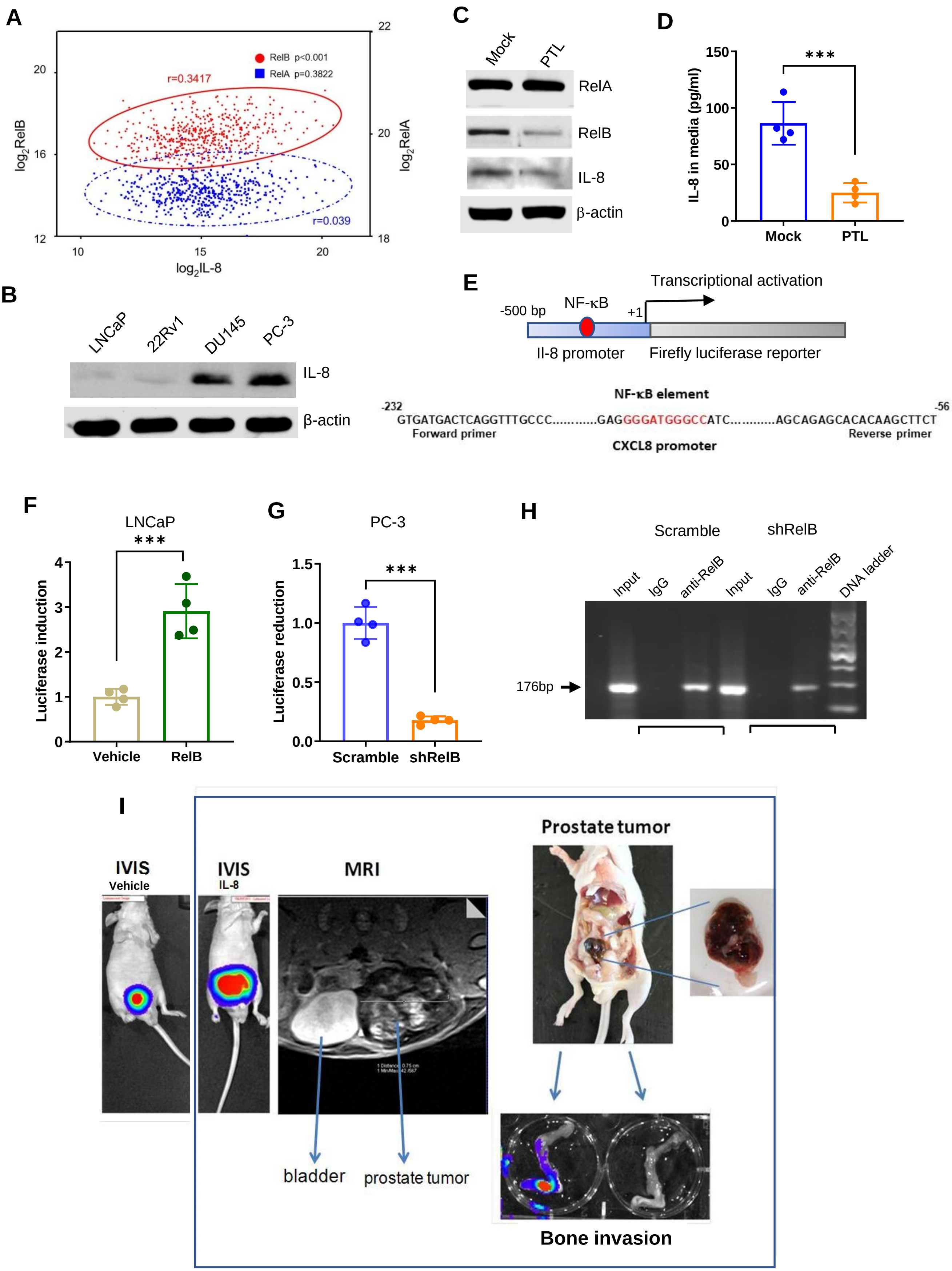
A
RelB p<0.001
RelA p=0.3822
r=0.3417
r=0.039
log2RelB
log2RelA
log2IL-8
B
LNCaP
22Rv1
DU145
PC-3
IL-8
β-actin
C
Mock
PTL
RelA
RelB
IL-8
β-actin
D
IL-8 in media (pg/ml)
Mock
PTL
E
Transcriptional activation
NF-κB
-500 bp
+1
Il-8 promoter
Firefly luciferase reporter
NF-κB element
-232
-56
GTGATGACTCAGGTTTGCCC..........GAGGGGATGGGCCATC..........AGCAGAGCACACAAGCTTCT
Forward primer
Reverse primer
CXCL8 promoter
F
LNCaP
Luciferase induction
Vehicle
RelB
G
PC-3
Luciferase reduction
Scramble
shRelB
H
Scramble
shRelB
Input
IgG
anti-RelB
Input
IgG
anti-RelB
DNA ladder
176bp
I
IVIS
Vehicle
IVIS
IL-8
MRI
Prostate tumor
bladder
prostate tumor
Bone invasion

Fig. S2. RelB-upregulated IL-8 to enhance PCa cell movability.

(A) The correlation of IL-8 to RelA or RelB in PCa tumor tissues was analyzed using the TCGA database. **(B)** The levels of IL-8 in AR-null cell lines (DU145 and PC-3) vs. AR-positive PCa cell lines (LNCaP and 22Rv1. **(C, D)** PLT inhibited IL-8 by suppressing RelB in PC-3 cells. **(E)** The human *CXCL8* promoter containing an NF-κB binding site was linked to the luciferase reporter. The sequence numbers indicated are according to the transcriptional start site as +1. **(F, G)** The effect of RelB on the transcriptional regulation of IL-8 in PCa cells was analyzed by quantifying the reporter response. **(H)** The *CXCL8* promoter was precipitated from PC-3 cell-derived chromatins using a RelB antibody and then quantified by PCR. Chromatins without pulldown served as an input control and pulled-down by IgG served as negative antibody control. **(I)** IL-8-overexpressed LNCaP cells were orthotopically implanted in mice prostates and formed tumors were imaged by IVIS and MRI**.** Thirty days after the cell implantation, tumor bone metastasis was examined. Data are shown as the mean ± SD. n=4, ***$p< 0.001$, t-test.

Figure S3

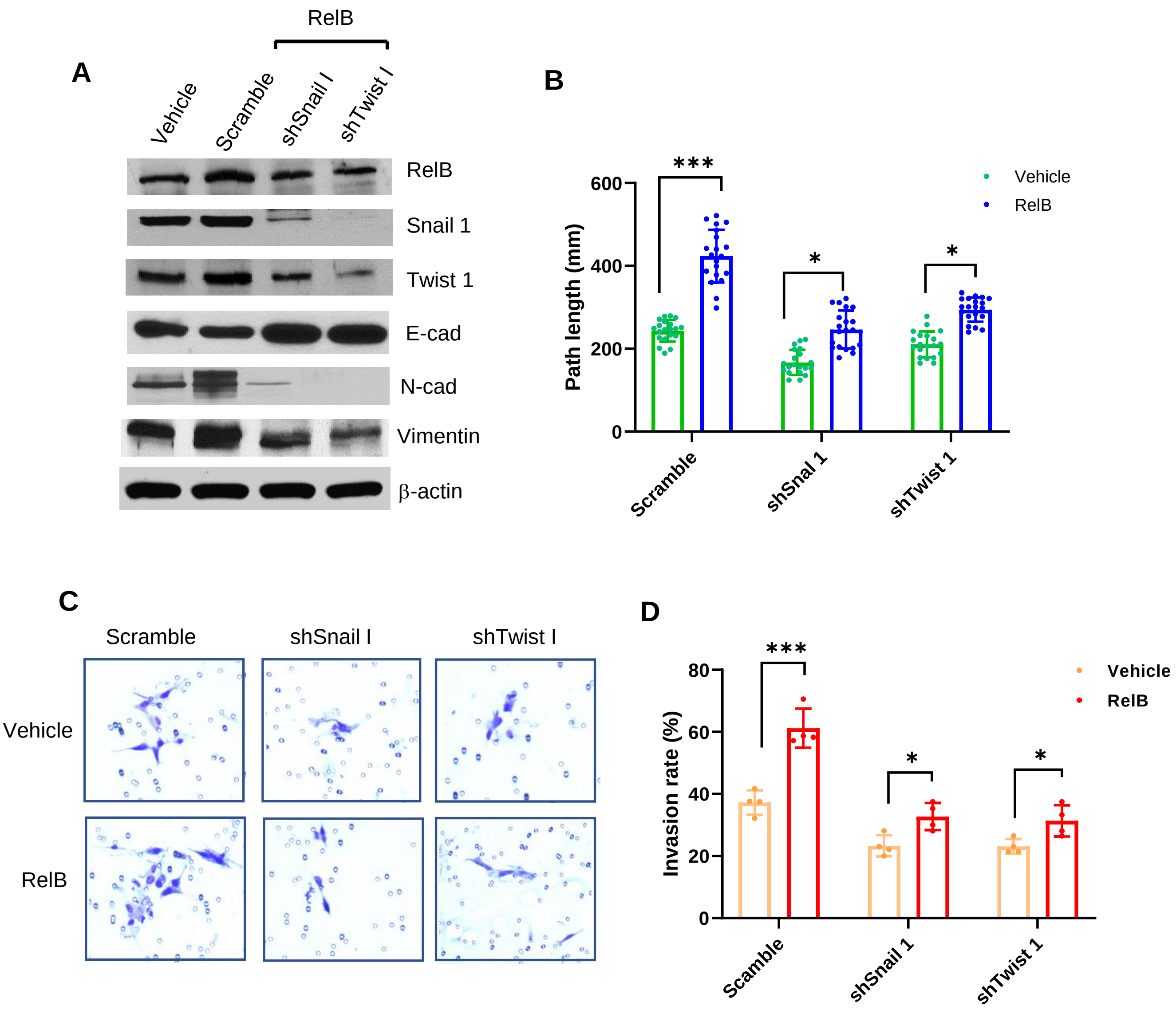

Fig. S3. The effect of RelB on Snail- and Twist-regulated EMT.

(**A**) Snail1 and Twist1 were silenced in RelB-overexpressed LNCaP cells, and the levels of EMT-associated proteins were quantified. (**B**) The relative cell migration was quantified by BioStation CT (n=20). (**C, D**) Cell invasion was analyzed using a transwell assay (n=4). Data are shown as the mean ± SD. *$p<0.05$, ***$p< 0.001$, t-test.

Figure S4

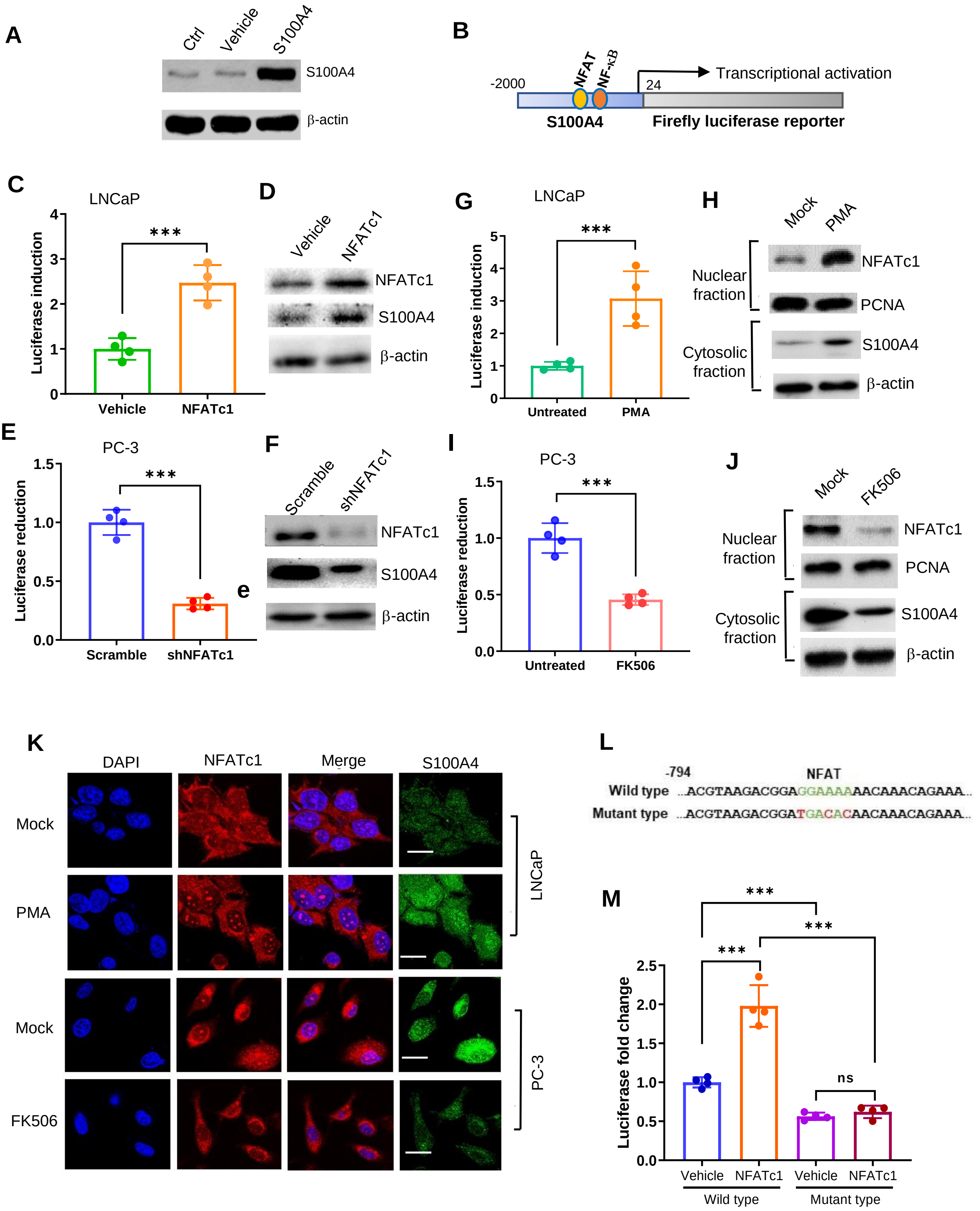
A
Ctrl
Vehicle
S100A4
S100A4
β-actin
B
-2000
NFAT
NF-κB
24
Transcriptional activation
S100A4
Firefly luciferase reporter
C
LNCaP
Luciferase induction
***
Vehicle
NFATc1
D
Vehicle
NFATc1
NFATc1
S100A4
β-actin
G
LNCaP
Luciferase induction
***
Untreated
PMA
H
Mock
PMA
Nuclear fraction
NFATc1
PCNA
Cytosolic fraction
S100A4
β-actin
E
PC-3
Luciferase reduction
***
Scramble
shNFATc1
F
Scramble
shNFATc1
NFATc1
S100A4
β-actin
e
I
PC-3
Luciferase reduction
***
Untreated
FK506
J
Mock
FK506
Nuclear fraction
NFATc1
PCNA
Cytosolic fraction
S100A4
β-actin
K
DAPI
NFATc1
Merge
S100A4
Mock
PMA
Mock
FK506
LNCaP
PC-3
L
-794
NFAT
Wild type ...ACGTAAGACGGAGGAAAAAACAAACAGAAA...
Mutant type ...ACGTAAGACGGATGACACAACAAACAGAAA...
M
Luciferase fold change
***
***
***
ns
Vehicle
NFATc1
Vehicle
NFATc1
Wild type
Mutant type

Fig. S4. The effect of NFAFc1 on the upregulation of S100A4.

(A) S100A4 was ectopically expressed in LNCaP cells, no transfection serves as a control**.** **(B-F)** The *S100A4* enhancer was linked to the luciferase reporter as indicated in (B); the sequence numbers are according to the transcriptional start site as +1. The effect of NFATc1 on S100A4 transcription was analyzed in NFATc1-overexpressed LNCaP cells (C, D), or NFATc1-silenced PC-3 cells (E, F). **(G, H)** PMA enhanced the reporter response in LNCaP cells by increasing NFATc1 nuclear levels. **(I, J)** FK506 repressed the reporter response in PC-3 cells by decreasing NFATc1 nuclear levels. **(K)** The effects of PMA and FK506 on NFATc1 nuclear translocation relevant to S10014 expression were analyzed by confocal microscopy. **(L)** The NFAT binding site in the *S100A4* gene was mutated as indicated. **(M)** The reporter response was quantified in NFATc1-overexpressed LNCaP cells. Data are shown as the mean ± SD. n= 4, ***$p < 0.001$, ns, no significance; t-test (C, G, E, I), one-way ANOVA (M). Scale bars, 20 μm in (K).

Figure S5

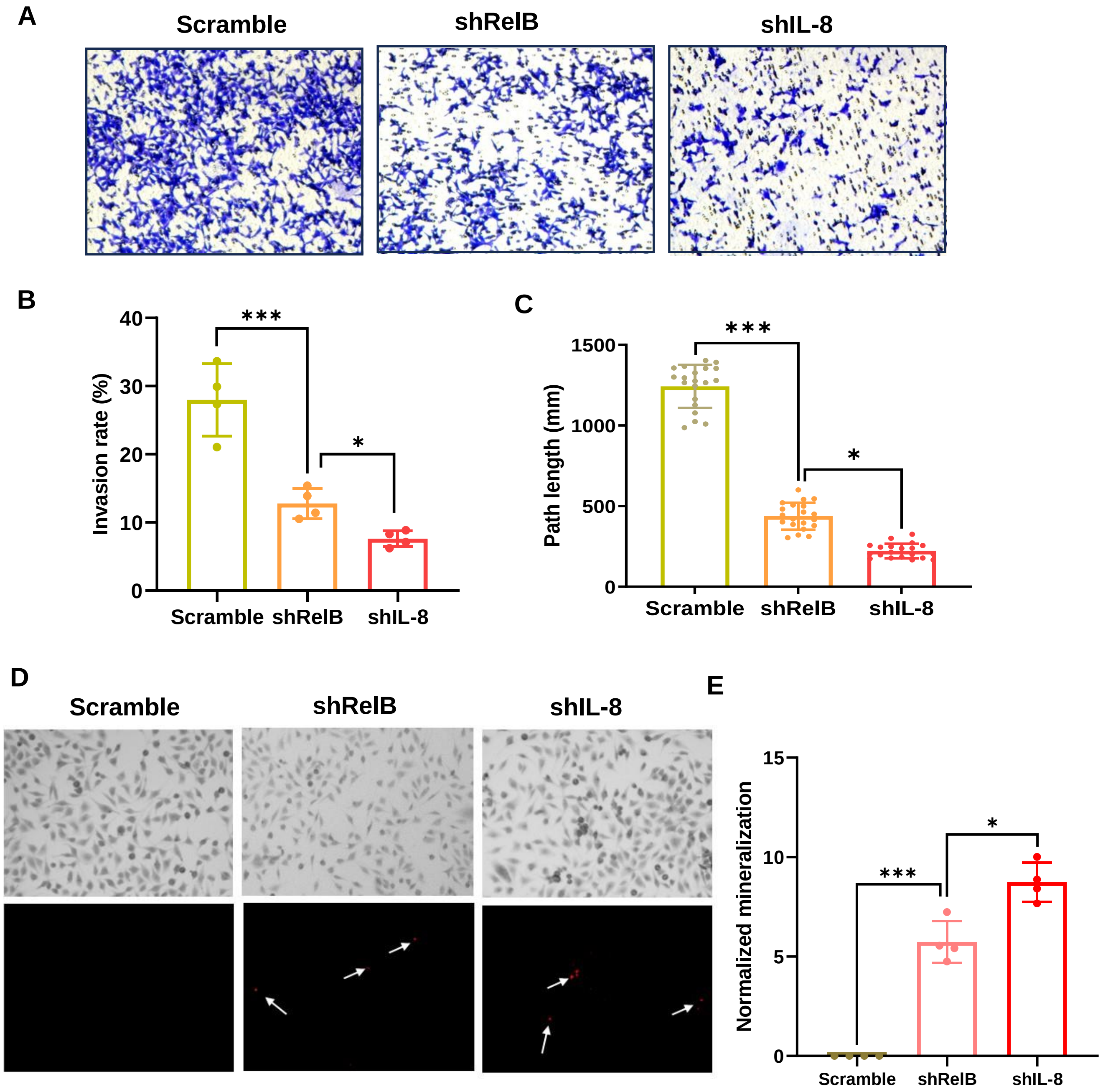

Fig. S5. The effect of RelB and IL-8 on PC-3 cell invasion, migration and mineralization.

(**A, B**) RelB and IL-8 were knocked down in PC-3 cells and cell invasion rate was analyzed with equal cell numbers using a transwell assay (n=4). (**C**) The cell migration was quantified by BioStation CT (n=20). (**D, E**) Cell mineralization was quantified using *in vitro* mineralization kit (n=4). Data are shown as the mean ± SD. *p<0.05, ***p< 0.001, t-test.

Figure S6

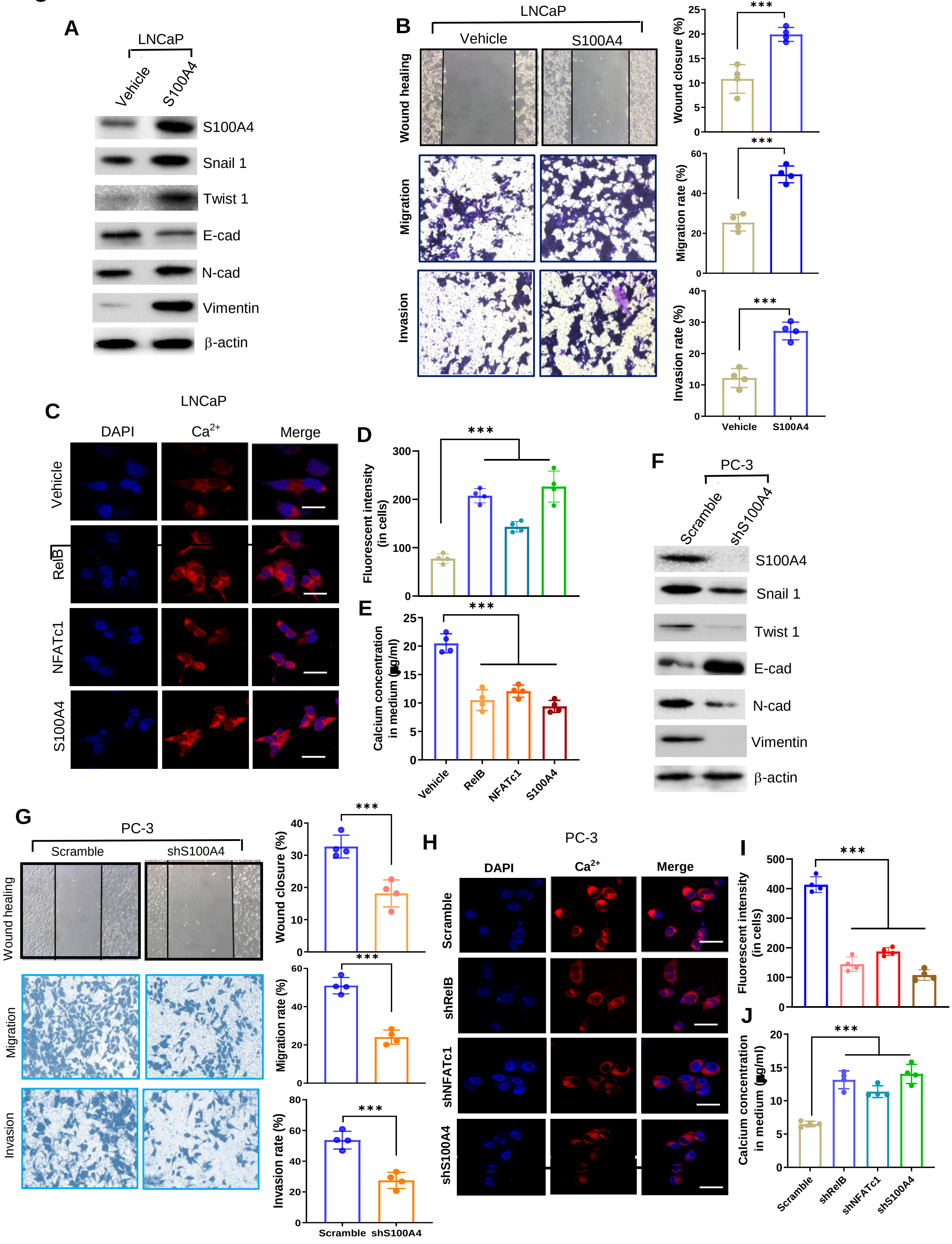

A
LNCaP
Vehicle
S100A4
S100A4
Snail 1
Twist 1
E-cad
N-cad
Vimentin
β-actin
B
LNCaP
Vehicle
S100A4
Wound healing
Migration
Invasion
Wound closure (%)
Migration rate (%)
Invasion rate (%)
***
Vehicle
S100A4
C
LNCaP
DAPI
Ca2+
Merge
Vehicle
RelB
NFATc1
S100A4
D
Fluorescent intensity (in cells)
***
E
Calcium concentration in medium (μg/ml)
***
Vehicle
RelB
NFATc1
S100A4
F
PC-3
Scramble
shS100A4
S100A4
Snail 1
Twist 1
E-cad
N-cad
Vimentin
β-actin
G
PC-3
Scramble
shS100A4
Wound healing
Migration
Invasion
Wound closure (%)
Migration rate (%)
Invasion rate (%)
***
Scramble
shS100A4
H
PC-3
DAPI
Ca2+
Merge
Scramble
shRelB
shNFATc1
shS100A4
I
Fluorescent intensity (in cells)
***
J
Calcium concentration in medium (μg/ml)
***
Scramble
shRelB
shNFATc1
shS100A4

Fig. S6. The effect of S100A4 on EMT and calcium homeostasis.

(**A**) The levels of EMT-associated proteins in S100A4-overexpressed LNCaP cells. (**B**) The relative cell mobility in S100A4-overexpressed LNCaP cells, as shown in wound closure, cell migration, and cell invasion. (**C, D**) Cellular calcium in RelB-, NFATc1-, or S100A4-overexpressed LNCaP cells were imaged and quantified using a specific fluorescence probe. (**E**) Free calcium in corresponding media was measured using a spectrophotometer. (**F**) The levels of EMT-associated proteins in S100A4-silenced PC-3 cells. (**G**) The effect of S100A4 on cell mobility in PC-3 cells. (**H-J)** The ratio of cellular calcium to free calcium in RelB-, NFATc1-, or S100A4-silenced PC-3 cells. Data are shown as the mean ± SD. n=4, ***p< 0.001; t-test (B, G), one-way ANOVA (D, E, I, J). Scale bars, 20 μm in (C) and (H).

Figure S7

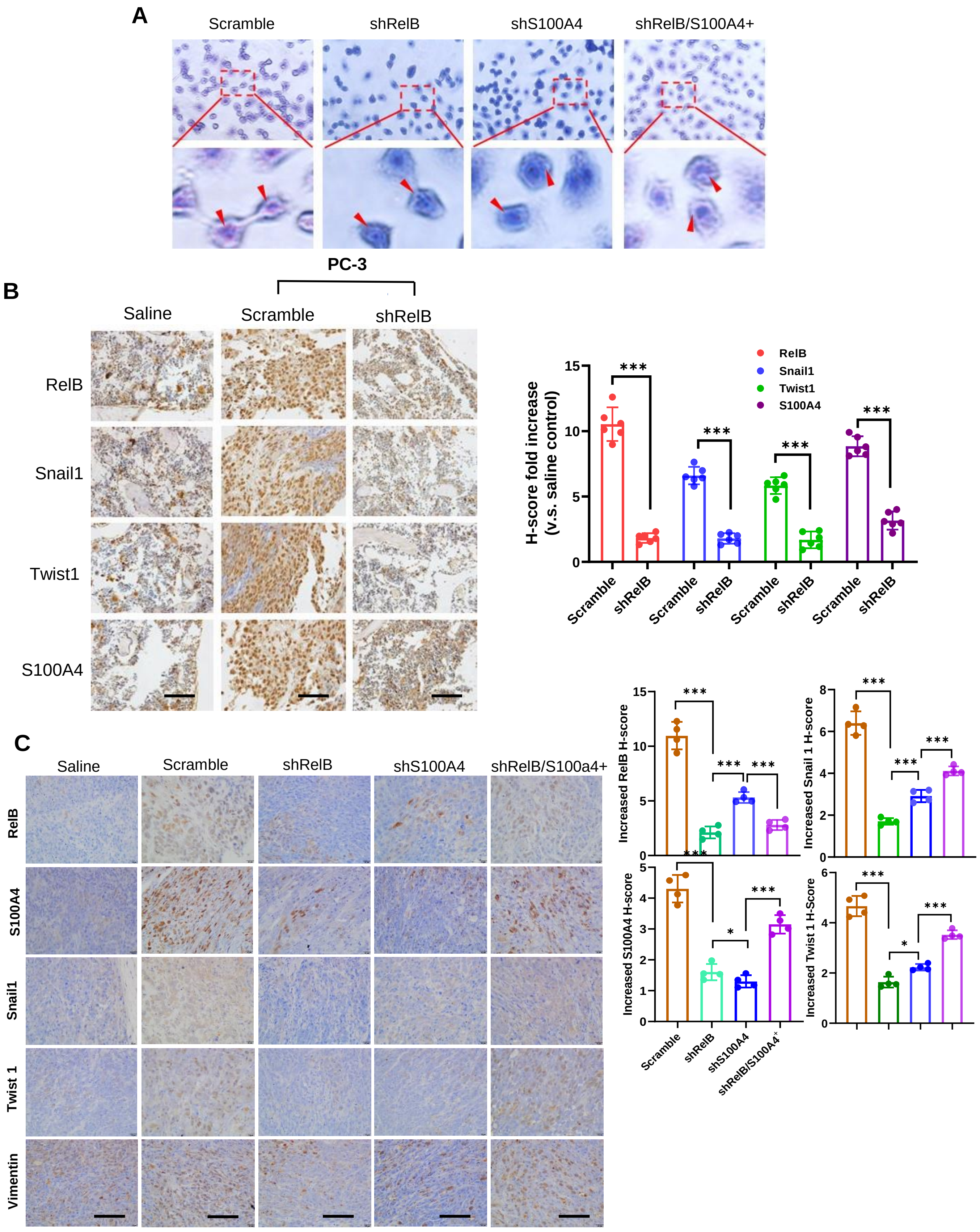

A
Scramble
shRelB
shS100A4
shRelB/S100A4+
PC-3
B
Saline
Scramble
shRelB
RelB
Snail1
Twist1
S100A4
H-score fold increase (v.s. saline control)
RelB
Snail1
Twist1
S100A4
***
C
Saline
Scramble
shRelB
shS100A4
shRelB/S100a4+
RelB
S100A4
Snail1
Twist 1
Vimentin
Increased RelB H-score
Increased Snail 1 H-score
Increased S100A4 H-score
Increased Twist 1 H-Score
Scramble
shRelB
shS100A4
shRelB/S100A4+

Fig. S7. Pathological analysis of osteolysis *in vitro* and *in vivo*.

(**A**) After stimulation by coculturing with PC-3 cells, the levels of tartrate-resistant acid phosphatase in RAW264.7 cells were imaged using a TRAP probe. (**B**) After intrathecally injecting RelB-depleted PC-3 cells into the knee articulations of mice, **t**he levels of RelB, Snail1, Twist1, and S100A4 in bone tissues were quantified by IHC (n=6). (**C**) After implanting RelB- S100A4-manipulated RM-1 cells into mice via the tail arteries, osteolytic metastasis-relevant protein levels in bone tissues by IHC (n-4). The fold changes in the H-scores were calculated by normalizing the images in controls. Data are shown as mean ± SD, *p<0.05, ***p< 0.001; t-test (B), one-way ANOVA (C). Scale bars, 50 μm in (B) and (C).

Figure S8

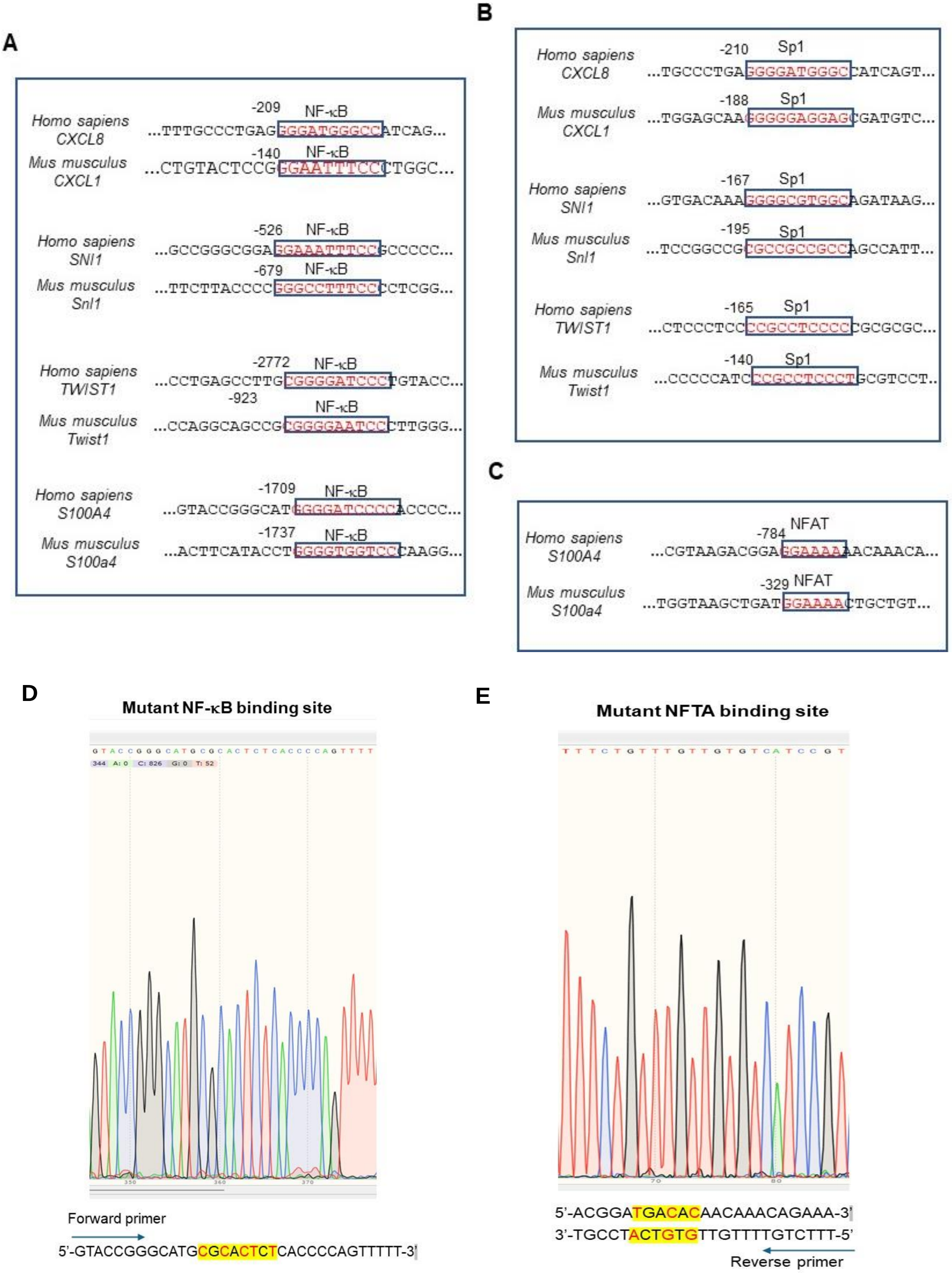
A
Homo sapiens CXCL8
-209 NF-κB
...TTTGCCCTGAGGGGATGGGCCATCAG...
Mus musculus CXCL1
-140 NF-κB
...CTGTACTCCGGGAATTTCCCTGGC...
Homo sapiens SNI1
-526 NF-κB
...GCCGGGCGGAGGAAATTTCCGCCCCC...
Mus musculus Snl1
-679 NF-κB
...TTCTTACCCCGGGCCTTTCCCCTCGG...
Homo sapiens TWIST1
-2772 NF-κB
...CCTGAGCCTTGCGGGGATCCCTGTACC...
-923 NF-κB
Mus musculus Twist1
...CCAGGCAGCCGCGGGGAATCCCTTGGG...
Homo sapiens S100A4
-1709 NF-κB
...GTACCGGGCATGGGGATCCCCACCCC...
Mus musculus S100a4
-1737 NF-κB
...ACTTCATACCTGGGGTGGTCCCAAGG...
B
Homo sapiens CXCL8
-210 Sp1
...TGCCCTGAGGGGATGGGCCATCAGT...
Mus musculus CXCL1
-188 Sp1
...TGGAGCAAGGGGGAGGAGCGATGTC...
Homo sapiens SNI1
-167 Sp1
...GTGACAAAGGGGCGTGGCAGATAAG...
Mus musculus Snl1
-195 Sp1
...TCCGGCCGCGCCGCCGCCAGCCATT...
Homo sapiens TWIST1
-165 Sp1
...CTCCCTCCCCGCCTCCCCCGCGCGC...
Mus musculus Twist1
-140 Sp1
...CCCCCATCCCGCCTCCCTGCGTCCT...
C
Homo sapiens S100A4
-784 NFAT
...CGTAAGACGGAGGAAAAAACAAACA...
Mus musculus S100a4
-329 NFAT
...TGGTAAGCTGATGGAAAACTGCTGT...
D
Mutant NF-κB binding site
Forward primer
5'-GTACCGGGCATGCGCACTCTCACCCCAGTTTTT-3'
E
Mutant NFTA binding site
5'-ACGGATGACACAACAAACAGAAA-3'
3'-TGCCTACTGTGTTGTTTTGTCTTT-5'
Reverse primer

Fig. S8. Validation of the NF-κB, Sp1, and NFAT elements in the relative genes.

(**A**) NF-κB elements in the relative human and murine genes. (**B**) Sp1 elements were found in the NF-κB-regulated human and murine genes. (**C**) NFAT elements in human and murine *S100A4* gene. The sequences of elements as indicated with red color in boxes. The sequence numbers are according to the transcriptional start site as +1. (**D, E**) NF-κB and NFAT binding sites in the human *S100A4* gene were mutated using a site-directed mutagenesis system and confirmed by DNA sequencing with a forward prime for the NF-κB site (D) and a reverse prime for the NFAT site (E).

Table S1. Primer sequences of RT-qPCR, ChIP and site-directed mutagen[illegible]

RT-qPCR		Sequence
S100A4		Forward: 5′-TGTGTCTTCCTGTCCTGCAT-3′<br>Reverse: 5′-CCCAACCACATCAGAGGAGT-3′
β-actin		Forward: 5′-CTACCTCATGAAGATCCTCACCGA[illegible]<br>Reverse: 5′-TTCTCCTTAATGTCACGCACGATT-[illegible]
**ChIP**	**Element**	**Sequence**
CXCL8	NF-κB	Forward: 5′-GTGATGACTCAGGTTTGCCC-3′<br>Reverse: 5′-AGAAGCTTGTGTGCTCTGCT-3′
Snail 1	Sp1	Forward: 5′-GAGACGAGCCTCCGATTGGC-3′<br>Reverse: 5′-AGGGCTTCCTGACGAGGAAA-3′
Twist I	Sp1	Forward: 5′-GTTGGGCGCTTTCTTTTTGG-3′<br>Reverse: 5′-TGGACACGTCCTGCATCATC-3′
Snail 1	NF-κB	Forward: 5′-AGTGATGTGCGTTTCCCTCG-3′<br>Reverse: 5′-TCGAGCGAAGCGAGGCCTCT-3′
Twist I	NF-κB	Forward: 5′-TGGGCAGGATGGTTCTGGTT-3′<br>Reverse: 5′-GCCAGGGGTCATTAGCCAGT-3′
S100A4	NF-κB	Forward: 5′-CTGACGTTCCTGAGGTGGTC-3′<br>Reverse: 5′-GCAACACAGAGGACCTTGTCT-3′
S100A4	NFAT	Forward: 5′-GGGCCAGATGTGCTAAAG-3′<br>Reverse: 5′-CAAGCCCACATCTCAGAAGCA-3′
**Mutagenesis**		**Sequence[a]**
NF-κB		Wild type: 5′-GTACCGGGCATGGGGATCCCCACCCCAGTTT-[illegible]<br>Mutant type: 5′-GTACCGGGCATGCGCACTCTCACCCCAGTTT-[illegible]
NFAT		Wild type: 5′-ACGTAAGACGGAGGAAAAAACAAACAGAAA-3′<br>Mutant type: 5′-ACGTAAGACGGATGACACAACAAACAGAAA-3′

[a]: the mutated nucleotides as indicated by underlines.
